# Quantifying the temporal trends of the combined effect of temperature and relative humidity on hand, foot, and mouth disease in Yunnan, China

**DOI:** 10.3389/fpubh.2025.1553278

**Published:** 2025-05-01

**Authors:** Zhaohan Wang, Yue Ma, Wennian Cai, Tao Zhang, Tian Huang, Tiejun Shui, Fei Yin, Haijun Yang

**Affiliations:** ^1^West China School of Public Health and West China Fourth Hospital, Sichuan University, Chengdu, China; ^2^National Cancer Center, National Clinical Research Center for Cancer, Cancer Hospital and Shenzhen Hospital, Chinese Academy of Medical Sciences and Peking Union Medical College, Shenzhen, China; ^3^Yunnan Center for Disease Control and Prevention, Kunming, China; ^4^Yan'an Hospital of Kunming City, Kunming, China

**Keywords:** HFMD, composite index, combined effect, temporal trends, time-varying DLNM

## Abstract

**Background:**

Hand, foot, and mouth disease (HFMD) poses a significant risk to children. While most studies focus on the individual effects of temperature or relative humidity, the combined effect of these factors and their temporal variations remain unclear. Understanding these effects is essential for designing effective public health interventions.

**Methods:**

Using daily meteorological and HFMD case data collected from 2010 to 2019 in 16 cities in Yunnan Province, China, we compared three composite indices (Humidex, heat index, and temperature–humidity index) to identify the indices that best captured the combined effect of temperature and humidity on HFMD risk. An extended time-varying distributed lag nonlinear model (DLNM) was used to examine how these effects shifted over time across population subgroups. Relative risk (RR) values at the 1%, 25%, 75%, and 99% quantiles were extracted to represent effects at extremely, moderately low, moderately, and extremely high levels.

**Results:**

The THI_a8_ demonstrated a monotonic upward exposure–response curve with narrower confidence intervals, more consistent relationships across cities, and the best model fit (Quasi-Akaike information criterion (QAIC) = 283564.2, Akaike information criterion (AIC) = 45.46, and Bayesian information criterion (BIC) = 62.30). HFMD risk decreased at extremely low (*RR* = 0.677, 95% CI: 0.632, 0.724) and moderately low THI_a8_ levels (*RR* = 0.766, 95% CI: 0.713, 0.823) but increased at moderately high (*RR* = 1.121, 95% CI: 1.084, 1.159) and extremely high THI_a8_ levels (*RR* = 1.478, 95% CI: 1.300, 1.680). Temporal analysis revealed a decreased HFMD risk at extremely low THI_a8_ values from 2010 to 2019, weakened protective effects at moderately low THI_a8_ values and an increased risk at extremely high THI_a8_ values. Subgroup analyses revealed that kindergarten children (3 ≤ age < 6 years) and females were particularly vulnerable.

**Conclusion:**

The THI_a8_ effectively captures the combined effect of temperature and relative humidity on HFMD risk revealing temporal variations. Adaptive public health strategies are needed to mitigate HFMD transmission under changing environmental conditions.

## 1 Introduction

Hand, foot, and mouth disease (HFMD) is an infectious disease primarily caused by enteroviruses and is widely distributed across the Asia-Pacific region (1–3). Most cases are slight and self-remitting, but severe cases can lead to complications such as aseptic meningitis, pulmonary edema, and myocarditis (4). Since the early 21st century, the Asia–Pacific region has experienced several large-scale HFMD outbreaks, with a noticeable increase in the proportion of severe cases, raising concerns among public health authorities (5, 6). In 2008, China designated HFMD a category C notifiable disease (7). Approximately 2 million cases are reported annually due to limited vaccination coverage (self-paid vaccination) and the lack of cross-protection against other serotypes causing HFMD (8, 9). HFMD has become the primary focus in the field of infectious diseases among children in China (10). The absence of specific antiviral treatments and high incidence rate make HFMD a substantial challenge to public health.

Previous studies have indicated that environmental factors, especially temperature and humidity, significantly increase the spread of HFMD (9–15). Several studies have analyzed the independent effects of temperature and humidity. However, the effects of these two factors on HFMD risk are not independent but rather have a mutual influence (14–19). This complex relationship cannot be fully captured by considering temperature or relative humidity alone.

Currently, two common approaches are applied to examine the combined effect of temperature and relative humidity, involving stratified or varying coefficient models (14–16). These methods allow researchers to examine how temperature affects HFMD risk at various humidity levels and vice versa, thereby clarifying their interaction. However, these methods are often relatively complex and may not effectively quantify how their combined effect influences disease outcomes. Another common approach involves constructing composite indices. Several indices have been explored for HFMD, including the heat index, temperature–humidity index (THI), and humidex (17–19). These indices provide a more comprehensive framework for assessing the combined effect of the two factors on HFMD risk, allowing for the use of exposure–response curves to quantitatively describe their relationship. This approach offers a more intuitive and simplified analysis of complex weather conditions and their impact on health outcomes. However, while these indices may offer a more robust framework for evaluating the effects of weather factors on disease risk, few studies have evaluated their suitability in characterizing the combined effect of these factors on HFMD risk.

Furthermore, several studies suggest that the effect of environmental variables on health risks evolves over time and are influenced by dynamic conditions such as climate change (20–26). For HFMD, the combined effect of temperature and relative humidity on disease outcomes may also change over time. Understanding how the combined effect of these two factors on HFMD risk evolves over time is crucial for accurately assessing current health risks and informing public health strategies. Such insights can help guide preventive measures, particularly in regions with unique climatic patterns like Yunnan Province.

This study focused on two key issues: determining which composite index best captures the combined effect of the two factors on HFMD risk and exploring how the combined effect changes over time. Our study area covered 16 cities in Yunnan Province, China. A two-stage time series analysis was applied to compare the performance of three composite indices, the heat index, THI, and Humidex, which were used to capture the combined effect of the two factors on HFMD risk. Using the optimal composite index, we conducted a time-varying DLNM to study the temporal trends of the combined effect of the two factors on HFMD risk in Yunnan, China, from 2010 to 2019. We also examined the differences in the time-varying combined effect across age and sex.

## 2 Methods

### 2.1 Study site

Located in China’s southwestern border area, Yunnan Province covers a total area of approximately 394,100 square kilometers (27). The province has complex climatic conditions characterized by diverse climate types and small annual temperature variations (28). The climate in Yunnan Province has periodic patterns, with meteorological factors showing complex and variable fluctuations over time. We selected all cities in Yunnan across cold, temperate, and tropical climates (including subtropical zones) as the study area (Supplementary Figures S1–S3).

### 2.2 Data sources

Data on the daily counts of HFMD cases were collected from the Yunnan Center for Disease Control and Prevention, covering the period from January 2010 to December 2019. This research examined HFMD cases in children under 6 years of age. To conduct the age subgroup analysis, we categorized the patients into two groups: Group 1 (0 ≤ age < 3 years) and Group 2 (3 ≤ age <6 years). The two groups specifically represent preschool children and kindergarten children.

Daily meteorological data were sourced from the National Meteorological Science Data Center,^1^ including daily average temperature (°C), average humidity (%), average wind velocity (m/s), sunshine duration (hours), daily rainfall (mm), and average air pressure (HPa). A comprehensive description of Yunnan’s meteorological monitoring stations can be found in Supplementary Table S1. To determine city-specific meteorological conditions, we averaged the daily variables for each city throughout the study period (Supplementary Table S2).

### 2.3 Description of composite indices

We included three widely discussed and applied composite temperature–humidity indices, namely, the Humidex, heat index, and THI, to assess their effectiveness in capturing the combined effect of these factors on HFMD risk. These composite indices have been previously explored in studies of HFMD and have demonstrated success in evaluating the combined effects of temperature and humidity on disease transmission (17–19). Their definitions and formulas are as follows.

#### 2.3.1 Humidex

The Humidex has been widely used as a composite index reflecting both temperature and relative humidity (29). The formula can be written as Equation 1:


(1) $$\mathrm{Humidex}=T+\frac{5}{9}\times \left[6.11\times 10^{\frac{7.5\times T}{237+T}}\times \frac{R}{100}-10\right]$$

T is the city-specific daily mean air temperature (°C), and R is the city-specific daily relative humidity (%). This index assumes a linear relationship where relatively high humidity amplifies the effect of temperature on human physiology, particularly under warm conditions. This index highlights how vapor pressure from relative humidity interacts with air temperature to increase perceived heat, making it a useful tool for understanding temperature–humidity interactions in moderate- to high-temperature environments.

#### 2.3.2 Heat index

The heat index uses a complex polynomial function to incorporate various factors (such as body height, weight, and sunlight exposure) that affect the heat dissipation of the body (30). The formula can be written as Equation 2:


(2) $$\begin{array}{l} \mathrm{Heat}\;\mathrm{index}=c_{1}+c_{2}T+c_{3}R+c_{4}TR+c_{5}T^{2}+c_{6}RH^{2}\;\\ \;+c_{7}T^{2}R+c_{8}TR^{2}+c_{9}T^{2}R^{2} \end{array}$$

This index incorporates nonlinear interactions, showing that the impact of humidity becomes more pronounced with increasing temperature. This index emphasizes how temperature and humidity jointly contribute to heat stress, with relative humidity playing a greater role under extreme heat conditions. The heat index is particularly suitable for capturing the dynamic interplay of these factors in hot and humid climates.

#### 2.3.3 THI

The THI is an alternative index developed by researchers (18). The THI accounts for the complex relationship between temperature and relative humidity, where the impact of humidity on the index value increases as the relative humidity deviates from 50%. The formula is as follows Equation 3:


(3) $$THI_{a}=T\times \omega^{\frac{R-50}{50}}$$

where T is the daily temperature (°C) and R is the daily relative humidity. ω represents the degree to which the impact temperature is greater at 100% relative humidity than at 50% relative humidity. On the basis of prior knowledge and sensitivity analysis results (Supplementary Table S9), we ultimately selected ω = 1.8 for the study. These findings indicate that 100% relative humidity increases the effect of temperature on HFMD risk 1.8 times more than 50% relative humidity does. Henceforth, we refer to this index as the THI_a8_.

### 2.4 Statistical analyses

A three-stage analysis was used to compare our candidate indices. The temperature was included as a reference for comparison. In the first stage, a DLNM with consistent model selection criteria was used to estimate the exposure–response relationships for each index across the 16 cities in Yunnan Province. In the second step, a multivariate meta-regression model was used to synthesize the first-stage results to evaluate the association patterns and model fit of each index, identifying the composite index that best captured the combined effect on HFMD risk. In the third stage, a time-varying DLNM meta strategy was used to analyze temporal variations in the combined effect on HFMD risk as the optimal composite index.

#### 2.4.1 Estimating the exposure–response relationship

The DLNM, developed by Gasparrini et al. (31), has been widely used in climatic and health-related time series. Owing to the sparse nature of HFMD case data in individual cities (Supplementary Table S3), we implemented a generalized DLNM using the quasi-Poisson distribution. Informed by prior knowledge (32) and sensitivity analysis results (Supplementary Text S1; Supplementary Figures S4–S10; Supplementary Tables S4–S9), the model was defined as follows Equation 4:


$$Y_{t}\sim \mathrm{Quasi}-\mathrm{poisson}\left(\mu_{t}\right)$$

(4) $$\begin{array}{l} \log\left(\mu_{t}\right)=\alpha +cb\left(M_{t},\mathrm{lag}\right)+\mathrm{Confounder}s_{t}+Auto_{t}\;\\ \;+\mathrm{ns}\left(time_{t},df\right)+\mathrm{Do}w_{t}+\mathrm{Holida}y_{t} \end{array}$$

where $Y_{t}$ represents the daily HFMD incidence on day_t_, $M_{t}$ represents different indices (Humidex, heat index, THI_a8_, and temperature as a reference), and $cb\left(\cdot \right)$ represents the cross-basis function. The model captures both the exposure-response and lag-response dimensions using a natural cubic spline (ns) with three equidistant knots for the exposure dimension and a natural cubic spline (df = 4) for the lag dimension. The lag period was defined as 0–14 days on the basis of the incubation and infectious periods of HFMD. Confounders represents the potential confounding variables, including sunshine duration, air pressure, wind velocity and relative humidity. Seasonal and long-term trends were adjusted via a natural cubic spline with 8 degrees of freedom per year. Auto represents autoregressive terms with data on a logarithmic scale, incorporating 1st- and 2nd-order lags. Dow represents the days of the week, and Holiday represents China’s public holidays. Dow was used to control for weekday effects and holiday effects.

#### 2.4.2 Evaluating the association patterns and model fit of the composite index

We aimed to identify a composite index that effectively characterizes the combined effect of temperature and humidity on HFMD risk, with certain desirable properties. First, the exposure–response curves derived from the index should exhibit monotonicity, consistency across different cities, and alignment with established epidemiological patterns. These properties will ensure the interpretability and practical utility of the index in public health interventions. Second, while association patterns provide critical epidemiological insights, better model fit enhances the reliability and robustness of the findings, offering a comprehensive basis for selecting the most suitable index.

To achieve this, we assessed both the association patterns and model fit of the three candidate indices. The performance of each index was assessed using the Quasi-Akaike information criterion (QAIC) (33), Bayesian information criterion (BIC) and Akaike information criterion (AIC). First, we aggregated the QAIC values from the DLNMs fitted to all 16 cities to assess the overall goodness-of-fit for each index across Yunnan Province. Second, a multivariate meta-regression model was applied to synthesize the city-level exposure–response relationships and examine the association patterns of each index at the provincial level. Finally, we compared the AIC and BIC values of the meta-regression models to identify the index with the best overall model fit, providing a comprehensive evaluation of its ability to represent the combined effect on HFMD risk.

#### 2.4.3 Exploring the temporal trends in the exposure–response association by using a time-varying DLNM

The Wald test can determine whether there is temporal heterogeneity in the associations for each city and overall (34). To assess the temporal trends in the combined effect on HFMD risk, we employed a time-varying DLNM (35). We added cross-basis time interactions to capture the time-varying relationships. The time-varying DLNM is as follows Equation 5:


$$Y_{t}\sim \mathrm{Quasi}-\mathrm{poisson}\left(\mu_{t}\right)$$

(5) logμt=α+cbMtlag+Confounderst+Autot+nstimet,df+Dowt+Holidayt+timet−reftime:cbMtlag

The terms and parameter settings are the same as those in the time-constant DLNM (2.4.1). $time_{t}$ represents the study time points. reftime represents the reference time point, which is the associated time we wanted to obtain. By changing the reference time, we could observe the associations at different time points. In this study, 2010, 2011, 2012, 2013, and 2019 were used as reference times to obtain the exposure–response curves for each corresponding year. To illustrate this temporal variation, we compared the exposure-response curves for 2010 and 2019, highlighting the changes in risk patterns across these years (34).

For each index, we selected the average value over the entire study period as a point of reference. To thoroughly examine the time-varying exposure–response relationships between each index and HFMD risk, we extracted RR values at the 1%, 25%, 75%, and 99% quantiles of the index values, which represented effects at extremely, moderately low, moderately, and extremely high levels, respectively.

Additionally, analyses stratified by sex and age were conducted to identify vulnerable groups, allowing us to assess variations in susceptibility across demographic groups and achieve a more detailed risk profile.

R (V.3.6.1), including the “dlnm,” “mvmeta,” “splines,” “tsModel,” “ThermIndex,” and “Weathermetrics” packages, was used for all the statistical analyses. Statistical significance was defined as a two-sided *p* value <0.05 for all tests.

## 3 Results

### 3.1 Epidemiological patterns of HFMD cases in Yunnan, China

**Figure 1:**
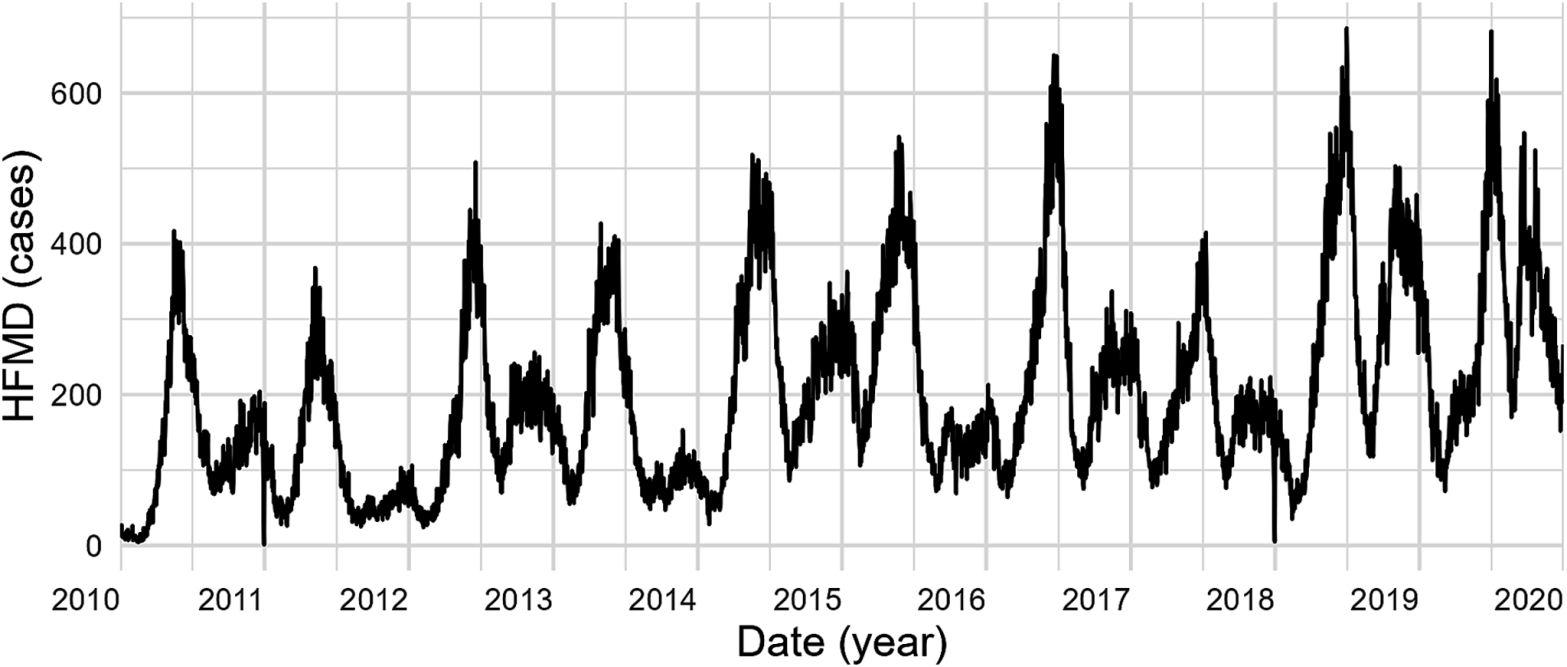
HFMD incidence trend in Yunnan Province, China: 2010–2019.

Between 2010 and 2019, 684,846 HFMD cases were reported across 16 cities in Yunnan, revealing a bimodal seasonal distribution with prominent peaks in both summer (April–July) and winter (October–December). Annually, case counts showed a significant upward trend, with the winter peak becoming increasingly pronounced (Figure 1). Detailed summary statistics of the characteristics are included in Supplementary Table S10.

### 3.2 Evaluating the association patterns and model fit of the composite indices

**Figure 2:**
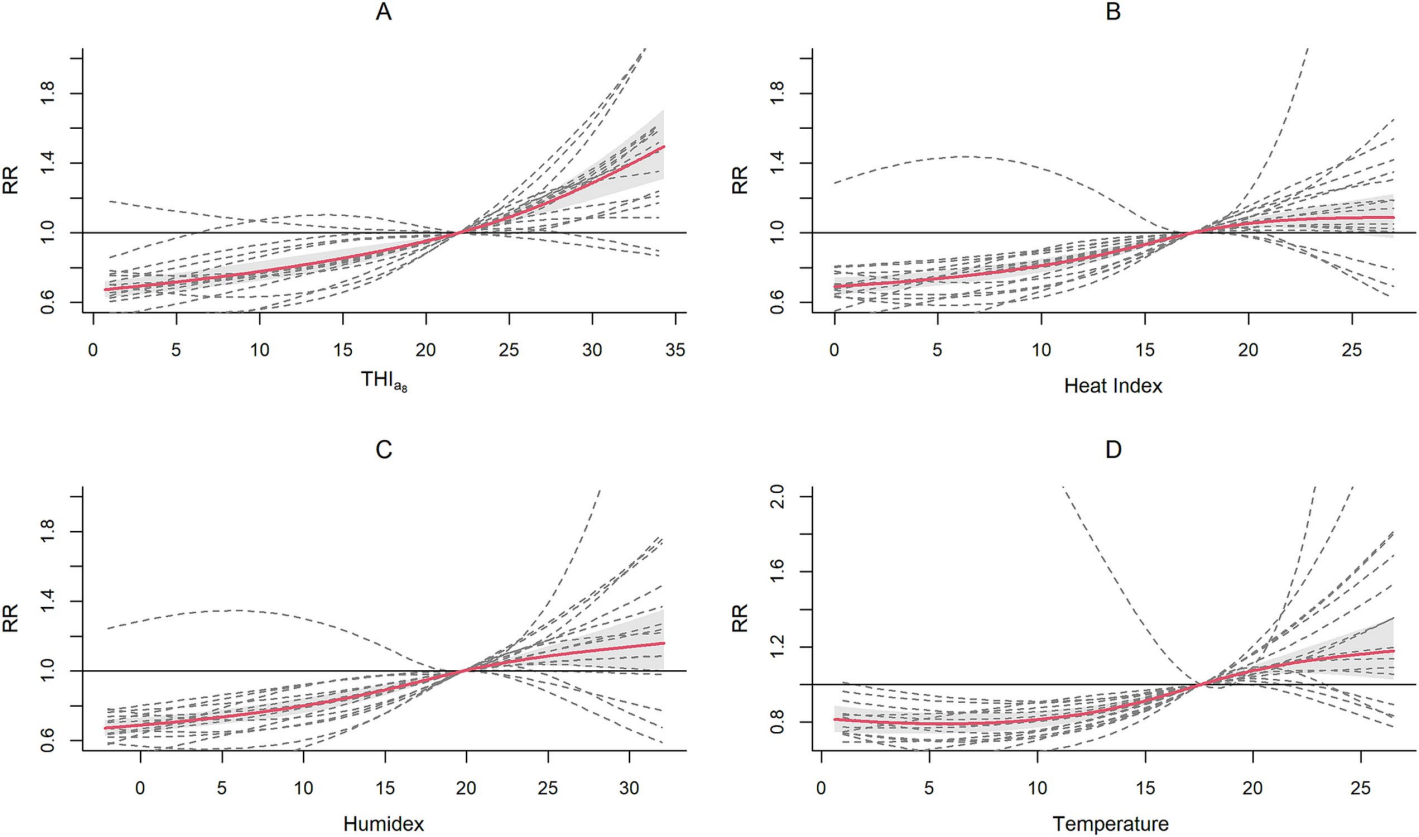
Association patterns for the THI_a8_ **(A)**, heat index **(B)**, Humidex **(C)**, and temperature **(D)**.

Figure 2 shows the cumulative exposure-response relationships across cities for each index, with the solid red line representing the pooled association and dashed gray lines illustrating city-specific variations. We found that the exposure–response curve for the THI_a8_ exhibited a consistent monotonically increasing trend, especially in the high-value range. This was accompanied by a narrower confidence interval than that of the other indices, indicating a more consistent exposure–response relationship across different cities. In contrast, the risk increasing trends for the heat index and Humidex begin to level off or even decrease in the high-value region, forming an “S”-shaped curve. These indices displayed wider confidence intervals, suggesting greater variability and less stable estimates across different cities.

The pooled analysis revealed a significant decrease in HFMD risk at extremely low and moderately low THI_a8_ levels but an increase at moderately high and extremely high levels. At extremely low THI_a8_ values, the *RR* was 0.677 (95% CI, 0.632, 0.724), which increased to 0.766 (95% CI, 0.713, 0.823) at moderately low THI_a8_ values. Conversely, the RR increased to 1.121 (95% CI, 1.084, 1.159) at moderately high THI_a8_ values and reached 1.478 (95% CI, 1.300, 1.680) at extremely high THI_a8_ values.

We subsequently compared the QAICs, AICs and BICs (Table 1). With respect to model fit, the THI_a8_ consistently had the best performance, regardless of whether the QAIC, BIC or AIC was considered. Considering both the association patterns and model fit, the THI_a8_ was more suitable for capturing the combined effect of the two factors on HFMD risk.

**Table 1:** The fit of the model for the different indices.

Indices	QAIC	AIC	BIC
THI_a8_	283564.2	45.46	62.30
Humidex	283687.8	48.39	65.23
Heat index	283757.1	46.29	63.13
Temperature	284925.4	57.50	74.34

### 3.3 Temporal trends in the cumulative exposure–response relationships between the THI_a8_ and HFMD risk

**Figure 3:**
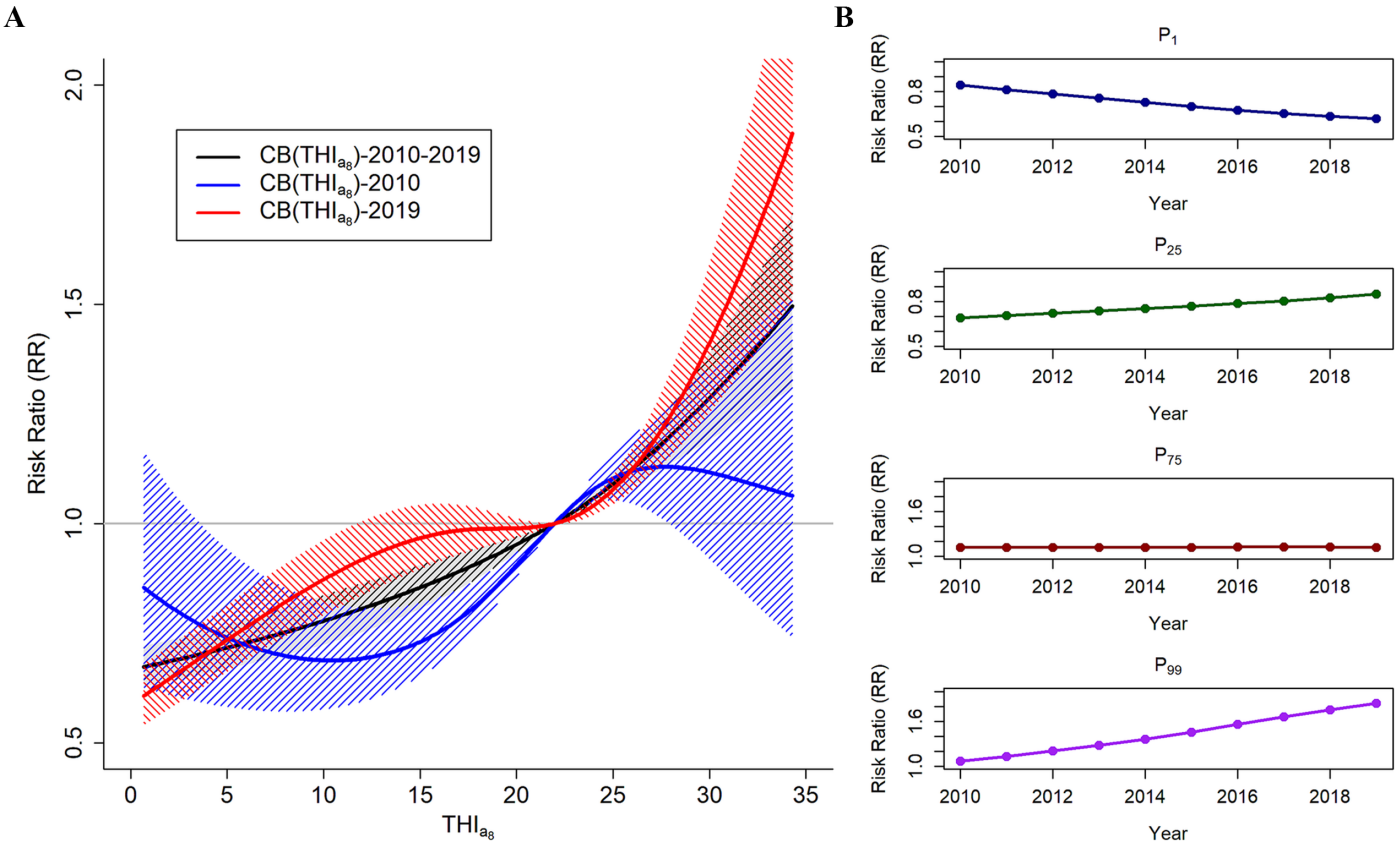
**(A)** Temporal trends in the associations between the THI_a8_ and HFMD risk in Yunnan Province from 2010 to 2019. **(B)** Temporal trends of the associations at P_1_, the 1st percentile of the THI_a8_; P_25_, the 25th percentile of the THI_a8_; P_75_, the 75th percentile of the THI_a8;_ and P_99_, the 99th percentile of the THI_a8._

The Wald test results revealed significant temporal trends across Yunnan Province and its individual cities (Supplementary Table S11). Figure 3A shows the effect of the THI_a8_ on HFMD risk in 2010 (blue) and 2019 (red). There was a significant difference in the effect of the THI_a8_ on HFMD risk between the beginning and end years of the study. These findings suggest that extremely low THI_a8_ values are linked to a lower risk of HFMD, whereas moderately low and extremely high THI_a8_ values correspond to an increased risk. The association between the THI_a8_ and changes in HFMD risk over time are shown in Supplementary Tables S12, S13. The annual variation in the effect of the THI_a8_ on HFMD risk during the study period is shown in Supplementary Figure S11.

Figure 3B and Table 2 illustrate the temporal trends in HFMD risk associated with varying levels of the THI_a8_ throughout the study period. At specific percentiles, the THI_a8_ values are as follows: 1.04 at P1, 9.10 at P25, 25.90 at P75, and 33.96 at P99. At extremely low THI_a8_ values (P1), the association with HFMD risk showed a consistent downward trend. By 2019, the estimated relative risk (RR) for the effect of the THI_a8_ had decreased to 0.617 (95% CI, 0.552, 0.691).

**Table 2:** Temporal trends in HFMD risk associated with low and high THI_a8_ levels in the first and last years of the study.

	Subgroups	2010–2019	2010	2019	2010–2019	2010	2019
		P_1_			P_25_		
Low THI_a8_	Total	0.677 (0.632,0.724)	0.842 (0.622,1.140)	0.617 (0.552,0.691)	0.766 (0.713,0.823)	0.691 (0.574,0.833)	0.850 (0.770,0.937)
	Male	0.659 (0.607,0.716)	0.867 (0.662,1.134)	0.603 (0.538,0.675)	0.757 (0.701,0.817)	0.706 (0.607,0.820)	0.844 (0.758,0.939)
	Female	0.667 (0.605,0.735)	0.831 (0.555,1.243)	0.593 (0.496,0.709)	0.748 (0.681,0.823)	0.655 (0.494,0.867)	0.842 (0.737,0.961)
	0 ≤ Age<3 years	0.667 (0.612,0.727)	0.965 (0.690,1.349)	0.568 (0.506,0.638)	0.738 (0.686,0.794)	0.720 (0.589,0.879)	0.798 (0.725,0.878)
	3 ≤ age<6 years	0.677 (0.613,0.748)	0.709 (0.510,0.986)	0.680 (0.570,0.813)	0.798 (0.740,0.859)	0.631 (0.501,0.793)	0.958 (0.851,1.079)
		P_75_			P_99_		
High THI_a8_	Total	1.121 (1.084,1.159)	1.118 (1.047,1.194)	1.119 (1.074,1.165)	1.478 (1.300,1.680)	1.068 (0.755,1.511)	1.846 (1.490,2.288)
	Male	1.132 (1.096,1.169)	1.121 (1.048,1.199)	1.125 (1.074,1.180)	1.548 (1.372,1.746)	1.098 (0.757,1.593)	1.914 (1.558,2.350)
	Female	1.136 (1.085,1.188)	1.130 (1.045,1.222)	1.133 (1.076,1.194)	1.534 (1.261,1.867)	1.038 (0.682,1.582)	1.996 (1.469,2.713)
	0 ≤ Age<3 years	1.131 (1.095,1.168)	1.182 (1.094,1.276)	1.108 (1.059,1.160)	1.465 (1.311,1.637)	1.394 (0.964,2.014)	1.668 (1.393,1.997)
	3 ≤ Age<6 years	1.130 (1.082,1.179)	1.041 (0.977,1.109)	1.148 (1.080,1.219)	1.647 (1.326,2.045)	0.704 (0.463,1.069)	2.426 (1.689,3.484)

P_1,_ 1st percentile of the THI_a8_; P_25_; 25th percentile of the THI_a8_; P_75_, 75th percentile of the THI_a8_; P_99_, 99th percentile of the THI_a8_.

In contrast, moderately low THI_a8_ (P_25_) values and extremely high THI_a8_ (P_99_) values were negatively associated. For moderately low THI_a8_ (P_25_) values, the protective effect steadily decreased, with the RR gradually approaching 1. Moreover, at extremely high THI_a8_ values (P_99_), the association with HFMD risk demonstrated a consistent upward trend. By 2019, the RR estimate for the effect of extremely high THI_a8_ values reached 1.846 (95% CI, 1.490, 2.288).

**Figure 4:**
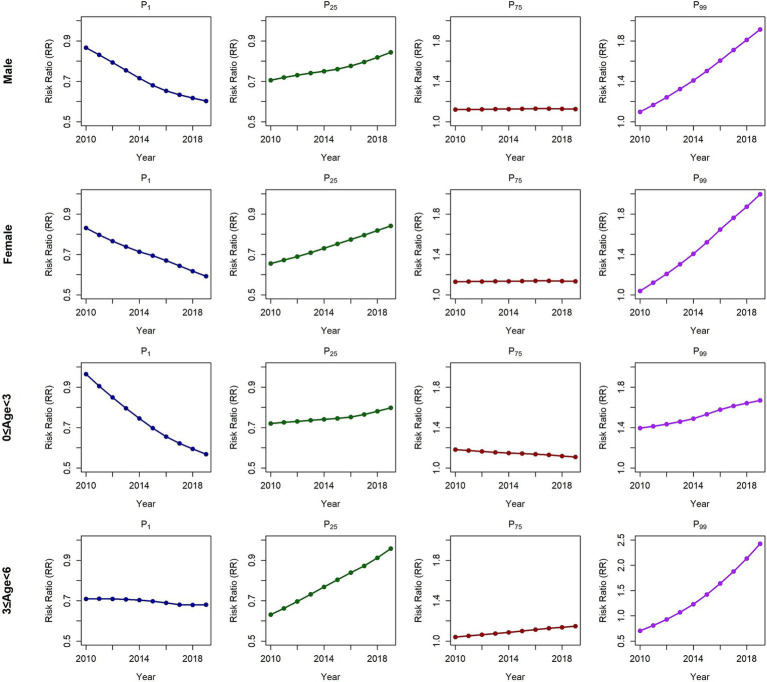
Temporal trends in the relationships between the THI_a8_ and HFMD risk among subgroups in Yunnan Province at the individual levels annually.

The subgroup analysis results revealed consistent findings (Figure 4; Table 2; Supplementary Tables S14–S21). Compared with that in 2010, the protective effect of extremely low THI_a8_ values in 2019 significantly increased across all subgroups, except the 3 ≤ age<6 subgroup. The risk reduction effect was evident among both sexes and other age groups. For the 3 ≤ age<6 group, we observed a marked weakening of the protective effect against HFMD at extremely low THI_a8_ values, which was more pronounced than that in the other subgroups. For extremely high THI_a8_ values, all subgroups experienced a substantial increase in HFMD incidence. Additionally, the female subgroup and the 3 ≤ age<6 subgroup presented a more pronounced increase in risk, whereas the male subgroup and the other age groups were comparatively less affected. Supplementary Figures S12–S15 provide detailed annual variations in THI_a8_ effects across subgroups, further illustrating these trends.

## 4 Discussion

To our knowledge, this is the first study to systematically analyze the temporal trends in the combined effect of temperature and relative humidity on HFMD risk using a comprehensive indice (THI_a8_). By evaluating the association patterns and model fit of three composite indices—the Humidex, the heat index, and THI_a8_—we found that the THI_a8_ effectively captured the combined effect of these two factors on HFMD risk. Our results also revealed temporal changes in the relationship between the THI_a8_ and HFMD risk, with a decreased risk at extremely low THI_a8_ values from 2010 to 2019, weakened protective effects at moderately low THI_a8_ values, and an increased risk at extremely high THI_a8_ values over time. Subgroup analyses further revealed that kindergarten-aged children (3 ≤ age < 6 years) and females were particularly vulnerable, stressing the need for targeted public health strategies.

The THI_a8_ quantifies the combined effects through a monotonic upward exposure–response curve. Specifically, HFMD risk decreased at low THI_a8_ levels but increases significantly at high THI_a8_ levels. The formula for the THI_a8_ quantifies how relative humidity affects the effect of temperature on HFMD risk. The results indicate that 100% relative humidity can increase the effect of temperature on HFMD risk by 1.8 times relative to 50% relative humidity. This pattern aligns with the results of previous studies, which indicate that warm and humid conditions contribute to enterovirus survival and replication, increasing HFMD risk (18, 32, 36–38). Specifically, both high-temperature and high-humidity conditions may increase HFMD risk by an additional 47.3% compared with nonhot or nonhumid conditions (38).

Furthermore, we identified significant temporal trends in the associations. Between 2010 and 2019, there was an increased protective effect at extremely low THI_a8_ values, a decreased protective effect at moderately low THI_a8_ values, and an increased risk at extremely high THI_a8_ values. Many studies support the notion that the associations between environmental factors and infectious diseases change over time (3, 20–24, 35, 39). A meta-analysis examining the drivers of global evolution and infectious disease risk highlighted the ongoing impact of environmental transformations—such as habitat degradation and biodiversity loss—on infectious disease dynamics (40). The study revealed that these changes alter the ecological landscapes of pathogens and vectors, resulting in changing associations between environmental factors and disease risk over time. Our research aligns with these findings.

The protective effect observed at extremely low THI_a8_ values may be partly due to climate-related factors. According to the THI calculation, extremely low THI_a8_ values are observed in cool and dry. The survival time of enteric viruses, such as EV-71 and CVA-16, is likely reduced, limiting viral transmission to susceptible hosts. This reduction in viral survival aligns with the lower RR values associated with extremely low THI_a8_ values. However, the observed strengthening of this protective effect over time could indicate that additional factors, such as public health interventions, are contributing to this trend. Specifically, strengthened HFMD epidemic warnings and prevention campaigns during autumn and winter may have further reduced the incidence of HFMD under these cooler and drier conditions. At moderately low THI_a8_ values, we observed a decreasing protective effect, with RR values approaching 1. A moderately low THI_a8_ value was associated with mild, stable conditions. These conditions neither inhibit nor significantly promote viral survival, creating an environment where HFMD-related pathogens such as EV-71 and CVA-16 can persist on surfaces and in water sources (37). Such conditions, often observed during transitional seasons such as spring and autumn, may increase the likelihood of transmission, particularly in densely populated settings with frequent close contact among children. During these mild weather periods, people may spend more time in semi-enclosed spaces—for example, schools might reduce outdoor activities to avoid sudden temperature changes, while households could keep windows closed for thermal comfort. This behavioral pattern might unintentionally increase indoor crowding, creating opportunities for person-to-person transmission even under seemingly low-risk meteorological conditions. Over time, the diminishing protective effect at this THI_a8_ highlights the need for proactive monitoring during these moderate conditions. At extremely high THI_a8_ values, we observed a steady increase in HFMD risk, with RR values increasing to 1.846 by 2019. This trend aligns with research suggesting that hot and humid conditions promote viral replication and spread (41). Extremely high THI_a8_ values correspond to hot, humid weather, which creates conditions favorable for viral transmission. Owing to intense daytime heat, children tend to participate in outdoor activities during cooler periods, such as early morning or late afternoon, increasing their exposure to contaminated environments and infected individuals in public spaces (42). Moreover, heat stress may weaken immune responses, heightening individuals’ susceptibility to infections and facilitating disease transmission (43). The increasing RR trend at extremely high THI_a8_ values over time may also be influenced by climate change. The increasing frequency of hot and humid conditions worldwide enhances viral stability and transmission, aligning with the observed increased HFMD risk (44, 45). In Yunnan Province, these shifts occurred against a backdrop of progressive climate changes, where meteorological observations indicate a gradual rise in annual mean temperature and increased frequency of extreme heat-humidity events during the study period (46).

Stratified analyses by age and sex highlighted population-specific susceptibilities. The 3 ≤ age<6 group had a lower sensitivity to the protective effects of extremely low THI_a8_ values and a greater risk to the effects of extremely high THI_a8_ values than did the 0 ≤ age<3 group. Preschool children’s (0 ≤ age<3) greater sensitivity to low THI_a8_ likely stems from their limited mobility and reliance on indoor environments where reduced viral persistence enhances protection. For older children, broader environmental exposure across homes, schools, and playgrounds dilutes these localized benefits. Under high THI_a8_, older children’s heightened risk aligns with emerging evidence that climate extremes amplify transmission through multiple pathways: warmer temperatures prolong viral survival in outdoor environments, heat stress weakens children’s mucosal defenses, and increased outdoor play elevates exposure risks (47). Females also presented a heightened risk at extremely high THI_a8_ values. This difference could be attributed to physiological differences between the sexes, particularly estrogen-mediated immune modulation that may delay viral clearance under heat stress and anatomical variations in sweat gland distribution that could impair evaporative cooling (48). Behavioral patterns may further amplify these physiological risks. For example, the frequent use of sun-protective attire (e.g., long sleeves, hats) among females likely increases hand-to-face contact frequency through repeated garment adjustments, which could elevate pathogen exposure risks.

Our results have practical implications in two key areas. First, the THI_a8_ effectively captures the combined effect of these two factors on HFMD risk. The dynamic impact on HFMD risk, as reflected by the THI_a8_, underscores the necessity of adaptive public health policies. Continuous monitoring of environmental conditions is essential to enable timely adjustments in prevention and public education efforts. For example, during periods of high temperature and humidity, heightened vigilance and targeted interventions while leveraging the protective effects of cool and dry conditions are crucial to mitigate HFMD risk. Second, focused interventions should be designed to address the specific vulnerabilities of different population subgroups. For example, public health campaigns aimed at reducing outdoor exposure during periods of extremely high THI_a8_ values could focus on children aged 3 to 5 years, as their increased outdoor activities make them more susceptible to HFMD. While our findings are grounded in Yunnan’s subtropical climate, the thermodynamic principles underlying THIa8’s humidity-modulated temperature effects suggest broader applicability. However, region-specific adaptations are needed—snow-cover periods in temperate zones may alter transmission dynamics through modified human-environment interactions. Our study has specific limitations. First, the ecological design inherently restricts causal inference due to unmeasured confounders like socioeconomic factors. As a result, the THI_a8_ is more suitable for characterizing HFMD risk patterns under varying temperature and humidity conditions rather than serving as a direct measure of the causal effect of these environmental factors on disease transmission. Second, the exclusion of viral serotype data (e.g., EV-A71 vs. CV-A16) requires caution, as pathogen-specific environmental responses may vary. However, laboratory-confirmed serotypes primarily came from severe/hospitalized cases, which may not fully represent environmental associations in the broader HFMD population. Third, although the THI_a8_ weight parameter (*ω* = 1.8) demonstrated statistical robustness, its biological interpretation requires validation through controlled viral persistence experiments.

## 5 Conclusion

Our study indicates that the impact of the combined effect, as reflected by the THI_a8_, on HFMD risk over time. Specifically, we observed that the protective effect at extremely low THI_a8_ values was increased, whereas the protective effect at moderately low THI_a8_ values was decreased. Concurrently, the risk associated with extremely high THI_a8_ values increased. These findings suggest that the effect of various environmental factors on disease risk is not static but rather dynamically changing. Therefore, to effectively meet future challenges and optimize public health strategies, continuous monitoring of environmental changes and their health impacts is essential. This ensures that prevention and response measures can be adapted in a timely manner.

## Data Availability

The data analyzed in this study is subject to the following licenses/restrictions: the datasets presented in this article are not readily available because the datasets generated and/or analyzed in this study are available from Yunnan Center for Disease Control and Prevention. The authors used the data for this current study under a license from the Yunnan CDC, so the data cannot be shared publicly. Requests to access these datasets should be directed to Yunnan Center for Disease Control and Prevention, https://www.yncdc.cn/.

## Footnotes

^1^ http://data.cma.cn/
