# Supplementary material for: Quantifying the temporal trends of the combined effect of temperature and relative humidity on hand, foot, and mouth disease in Yunnan, China
Source: Front Public Health. 2025 May 1;13:1553278. doi: 10.3389/fpubh.2025.1553278 (PMC12078327; doi:10.3389/fpubh.2025.1553278)
Supplement: Supplementary file 1 [file Data_Sheet_1.PDF]

Supplementary Material

Quantifying the Temporal Trends of the Combined Effect of Temperature and Relative Humidity on Hand, Foot, and Mouth Disease in Yunnan, China

Zhaohan Wang [1,¶], Yue Ma [1,¶], Wennian Cai[2],Tao Zhang[1], Tian Huang[3],Tiejun Shui[3], Fei Yin [1,*], Haijun Yang[4,*]

*** Correspondence:**

Email:82174831@qq.com(Fei Yin), yyanghaijun@126.com(Haijun Yang)

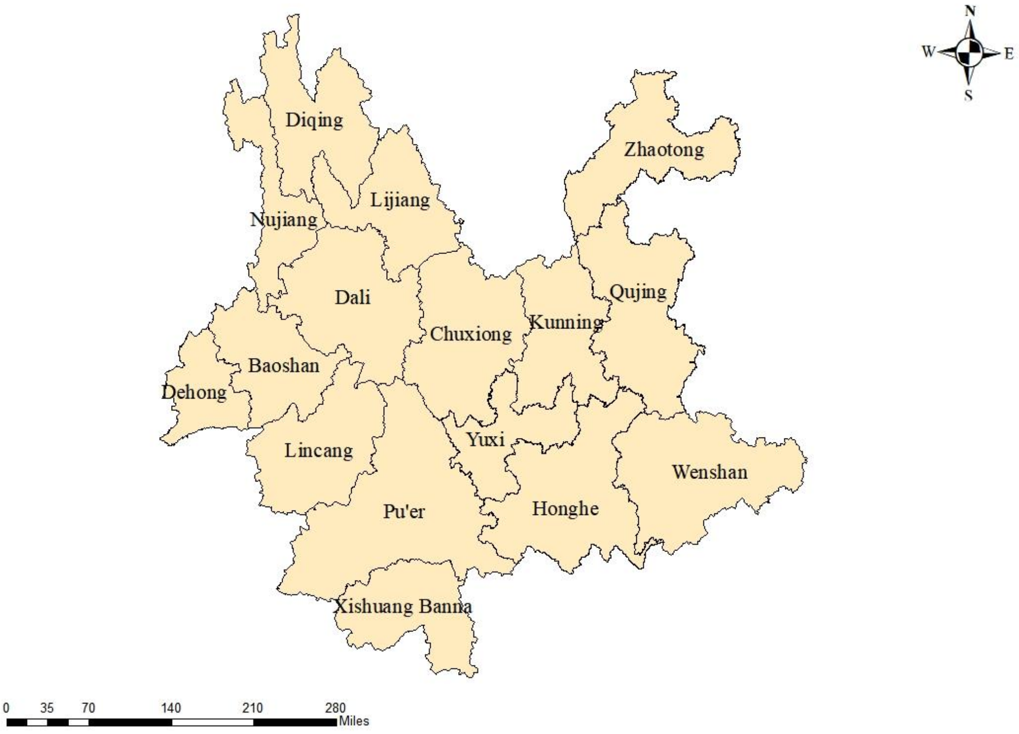

Supplementary Figure S1. Map of the Administrative Divisions of Yunnan Province

Supplementary Table S1. Meteorological monitoring station of Yunnan Province

Code	Point	Latitude	Longitude
56444	Deqin	28.483	98.917
56533	Gongshan	27.75	98.667
56543	Shangri-La	27.833	99.7
56548	Weixi	27.167	99.283
56643	Liuku	26.867	98.85
56651	Lijiang	26.85	100.217
56664	Huaping	26.633	101.267
56684	Huize	26.4	103.25
56739	Tengchong	24.983	98.5
56748	Baoshan	25.117	99.183
56751	Dali	25.7	100.183
56778	Kunming	25	102.65
56856	Jingdong	24.467	100.867
56875	Yuxi	24.333	102.55
56886	Luxi	24.533	103.767
56946	Gengma	23.55	99.4
56950	Shuangjiang	23.467	99.8
56951	Lincang	23.883	100.083
56954	Lancang	22.567	99.933
56964	Simao	22.783	100.967
56966	Yuanjiang	23.6	101.983
56969	Mengla	21.467	101.583
56977	Jiangcheng	22.583	101.85
56991	Yanshan	23.617	104.333
59007	Guangnan	24.067	105.067
56586	Zhaotong	27.35	103.717

Supplementary Table S2. City-level meteorological conditions of 16 cities in the Yunnan (daily data calculating by arithmetic mean)

City	Temperature (°C)	Relative humidity (%)	Wind velocity (m/s)	Sunshine hours (h)	Precipitation (mm)	Pressure (hpa)
Kunming	15.56	68.55	2.66	6.42	2.41	806.67
Qujing	14.49	71.67	2.56	5.82	2.24	806.68
Yuxi	19.85	67.59	2.04	6.19	2.11	878.33
Baoshan	17.02	68.89	1.66	6.64	2.77	835.21
Zhaotong	12.13	73.58	1.69	4.77	1.93	798.29
Lijiang	14.13	59.36	2.50	6.78	2.44	772.72
Pu’er	20.18	71.11	1.22	6.41	3.12	883.17
Lincang	18.47	70.29	1.20	6.40	3.04	858.63
Chuxiong	18.06	66.37	2.00	6.44	2.57	843.00
Honghe	19.08	73.67	2.24	5.74	2.76	873.69
Wenshan	17.60	78.36	1.97	4.70	2.91	864.68
Xishuang Banna	20.95	79.40	0.98	5.71	4.38	908.90
Dali	16.17	65.16	2.08	6.09	2.49	808.59
Dehong	16.81	76.33	1.55	6.45	4.02	850.73
Nujiang	18.97	67.55	1.28	4.99	2.69	879.58
Diqing	7.51	67.44	1.99	5.47	1.93	696.59

Supplementary Table S3. Over-dispersion test for the HFMD cases time series of each city

City	Statistics	*P*-value
Kunming	161818.30	<0.05
Qujing	85999.00	<0.05
Yuxi	46273.60	<0.05
Baoshan	32437.81	<0.05
Zhaotong	25308.96	<0.05
Lijiang	22379.97	<0.05
Pu’er	38748.71	<0.05
Lincang	23858.43	<0.05
Chuxiong	34350.31	<0.05
Honghe	51334.01	<0.05
Wenshan	32784.20	<0.05
Xishuang Banna	25075.40	<0.05
Dali	34972.17	<0.05
Dehong	29964.19	<0.05
Nujiang	11441.67	<0.05
Diqing	12846.77	<0.05

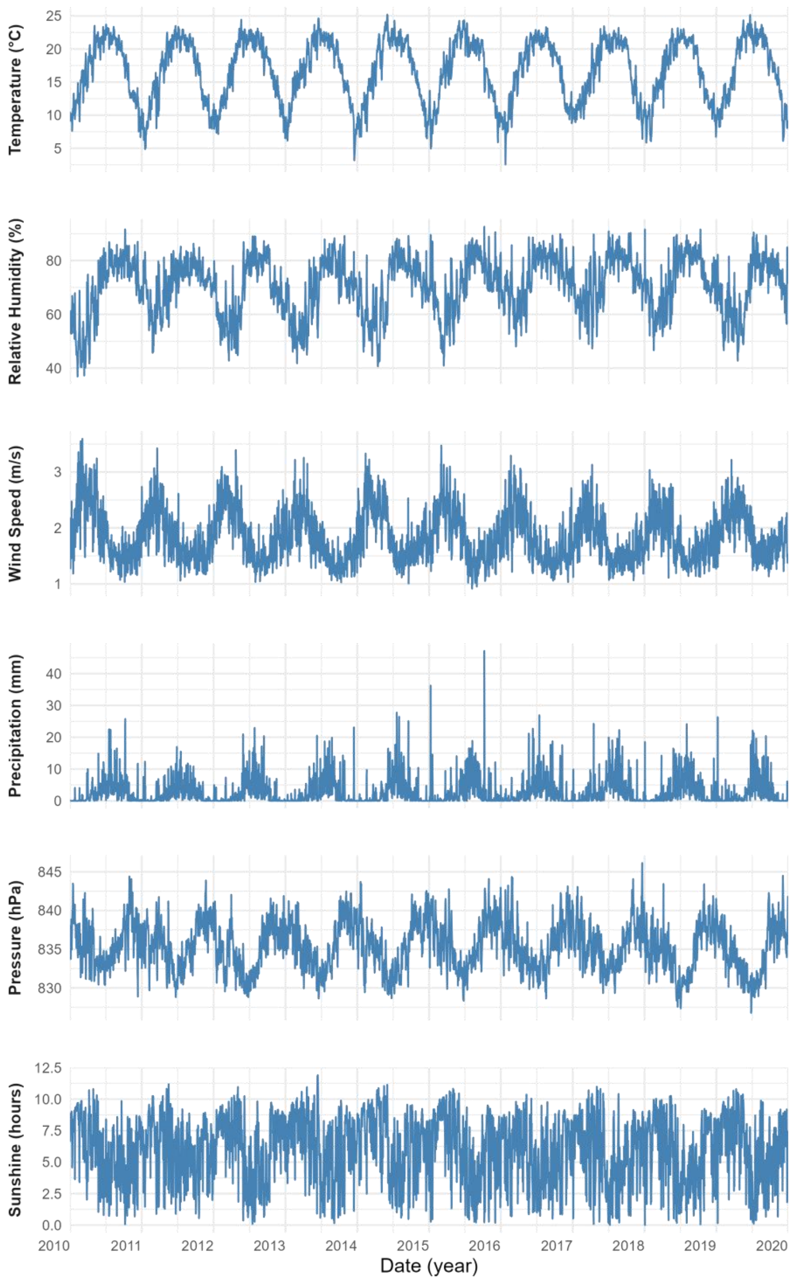

Supplementary Figure S2. Daily trends of meteorological variables in Yunnan Province (2010-2019).

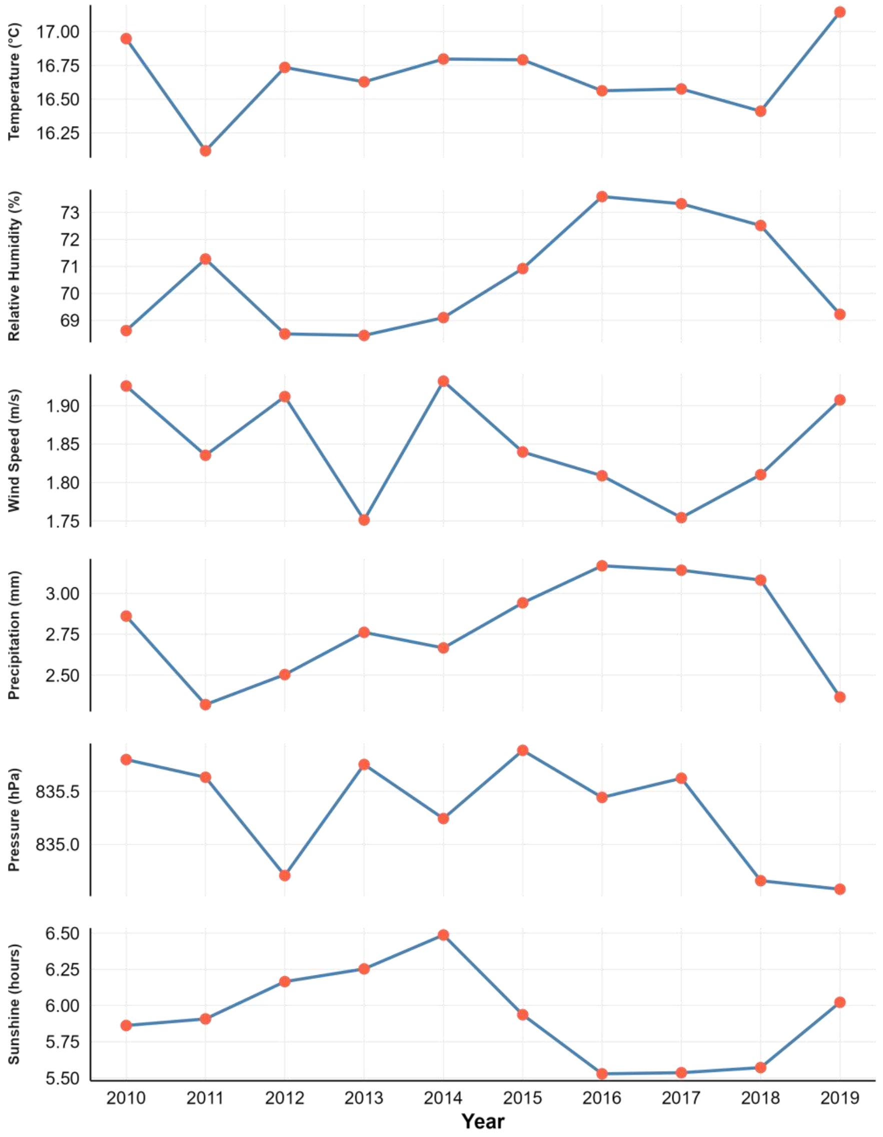

Supplementary Figure S3. Annual trends of meteorological variables in Yunnan Province (2010-2019).

Supplementary Text S1. Sensitivity Analysis (Take temperature as example)

This study used a systematic analysis approach to set parameters for the DLNM model based on prior knowledge. Parameters were evaluated by analyzing the average of estimates (AOE) and the total quasi-Akaike information criterion (QAIC). AOE assessed whether parameter selection captured estimate biases, while QAIC total evaluated improvements in model fit. Parameters were determined by considering model simplicity and rationality, integrating AOE and QAIC results.

1. Selecting the degrees of freedom (df) to manage long-term trends and seasonal patterns.

We used a natural cubic spline (ns) with degrees of freedom (df) varying from 1 to 10 per year to manage long-term trends and seasonality. According to **Figure S4**, QAICs and AOE stabilized when the df reached 8 per year. Therefore, we settled on 8df/year for controlling these patterns.

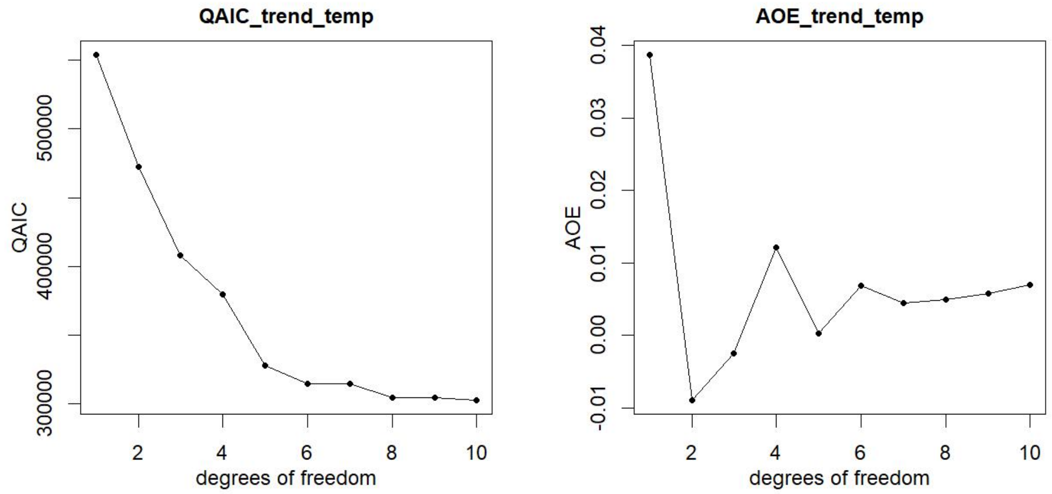

Supplementary Figure S4. The QAICs (left panel) and AOE (right panel) of the fitted model by changing the degrees of freedom of natural spline.

2.Selecting the form of confounders.

Based on previous studies, we included sunshine duration, relative humid, wind velocity, pressure and rainfall as confounding variables. Using a reference model without confounders, we built eight models for each variable (**Table S4**). From the combined results, we selected model C2 for sunshine duration, C6 for relative humid, wind velocity and pressure, C8 for rainfall. For detailed information, refer to **Supplementary Figure S5-S8**.

Supplementary Table S4. The parameter settings of confounder

Notation	Association	Lag days	Forms of lag days
C0	-	-	-
C1	Linear	4	-
C2	Linear	4-10	SMA
C3	Linear	4-10	EMA
C4	Linear	4-10	ns with 4 df
C5	Nonlinear	4-10	-
C6	Nonlinear	4-10	SMA
C7	Nonlinear	4-10	EMA
C8	Nonlinear	4-10	ns with 4 df

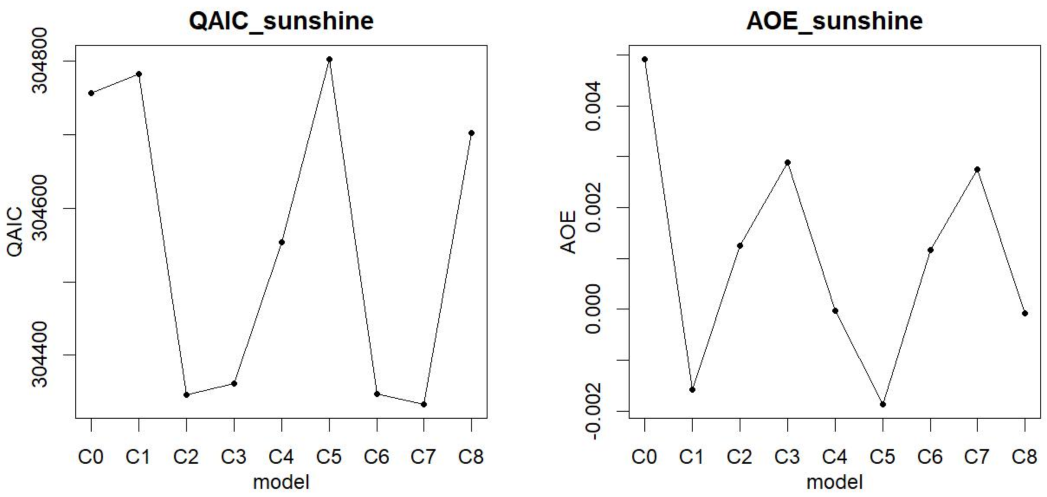

Supplementary Figure S5. The QAICs (left panel) and AOE (right panel) of the fitted model of sunshine duration.

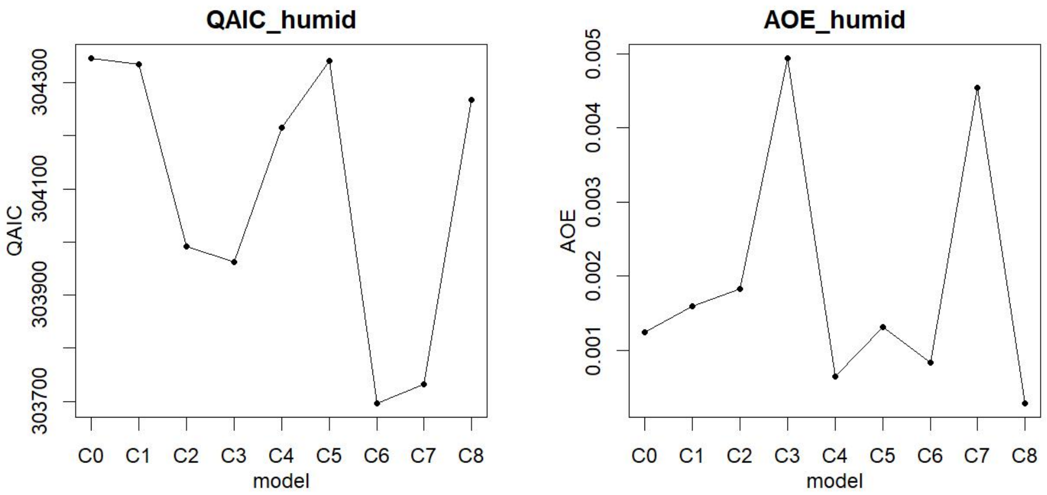

Supplementary Figure S6. The QAICs (left panel) and AOE (right panel) of the fitted model of relative humid.

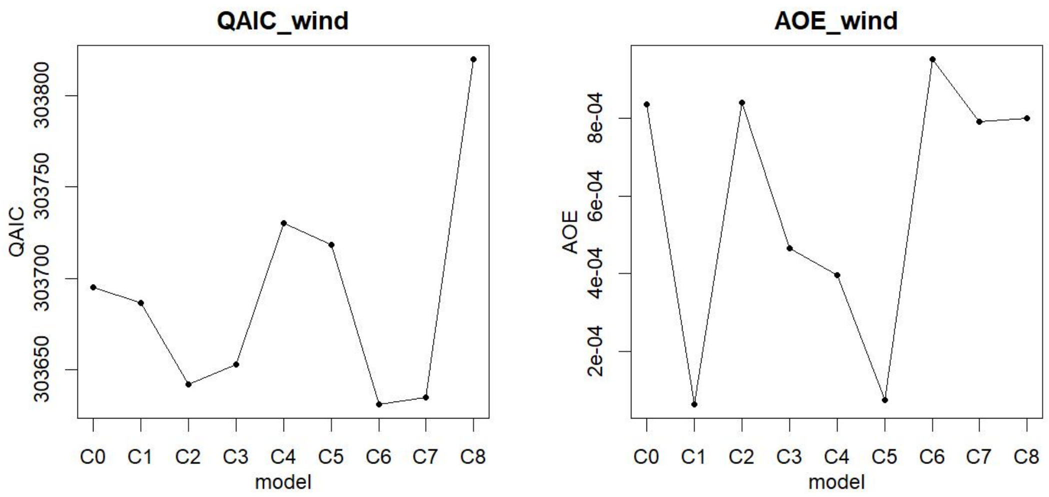

Supplementary Figure S7. The QAICs (left panel) and AOE (right panel) of the fitted model of wind.

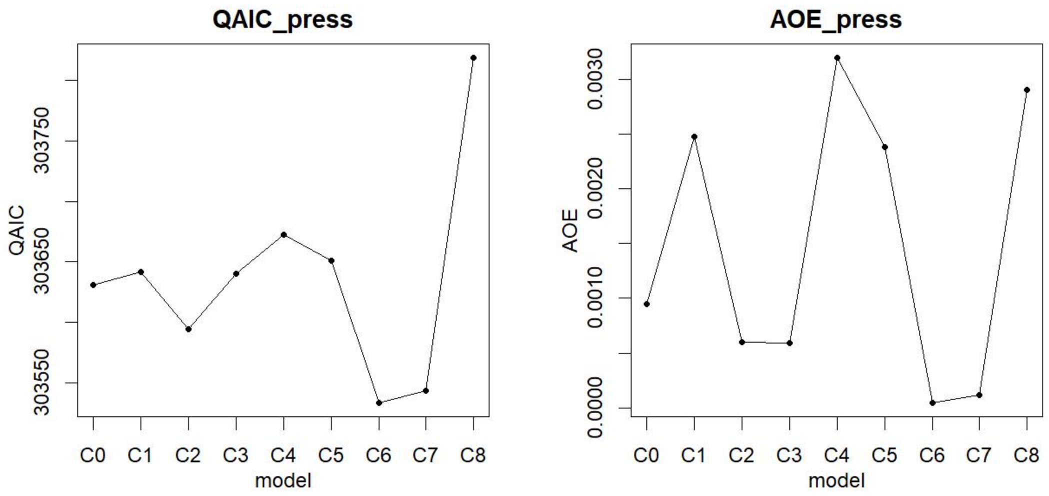

Supplementary Figure S8. The QAICs (left panel) and AOE (right panel) of the fitted model of press.

3. selecting the autoregressive term.

When setting the autoregressive terms, we considered various lag orders and forms of inclusion. Using a model without autoregressive terms as the reference, we established six models (**Table S5**). Based on the lowest QAIC values, model **A2** was selected for the autoregressive term.Shown as **Supplementary Figure S9**.

Supplementary Table S5. The parameter settings of autoregressive term.

Notation	Lag	Scale	forms
A1	Lag 1~2	Original scale	None
A2	Lag 1~2	Logarithmic scale	None
A3	Lag 1~2	Logarithmic scale	SMA
A4	Lag 4~10	Original scale	None
A5	Lag 4~10	Logarithmic scale	Ns(df=4)
A6	Lag 4~10	Logarithmic scale	SMA

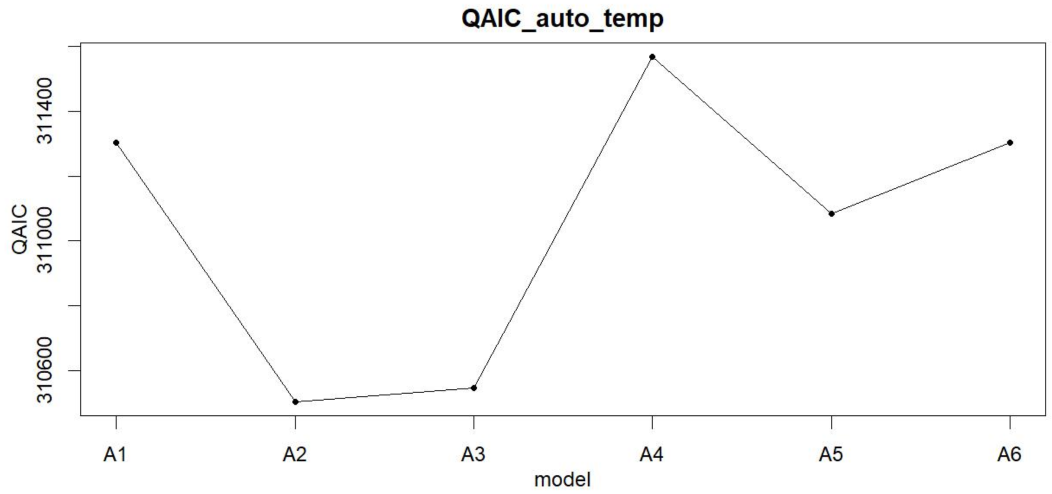

Supplementary Figure S9. The QAICs of the fitted model of autoregressive term.

4. selecting the lag structure of temperature on HFMD

To thoroughly investigate the structure of lag effects, we utilized unrestricted lag distribution models across 16 cities in the Yunnan province, systematically estimating the effects of temperature over lag days ranging from 0 to 30. Based on the average of estimates (AOE) depicted in Figure S10, the incubation period (3-5 days), and the prolonged infectious period (nearly 2 weeks) of HFMD, as well as the optimized model performance (**Supplementary table S6**), we identified a lag period of 0 to 14 days for this study. Moreover, the lag-response relationships were modeled using a natural cubic spline (ns) function, with options of 3 to 5 degrees of freedom (df). Ultimately, we selected a natural cubic spline with 4 df for the lag distribution of all variables.shown as **Supplementary Figure S10, Supplementary table S6 and Supplementary table S7.**

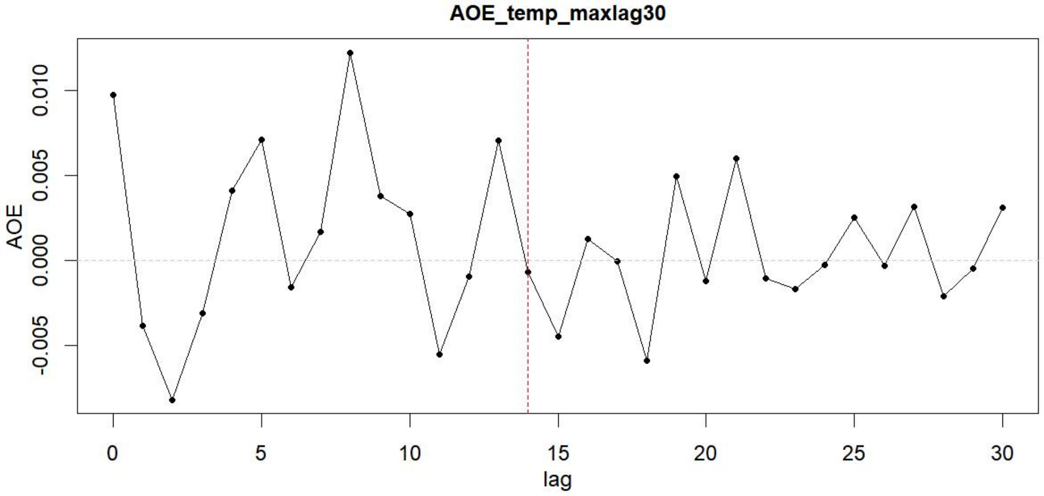

Supplementary Figure S10. The AOE of lag days of the fitted model

Supplementary Table S6. The QAICs of lag days of the fitted model.

Max lag days	*QAICS*
7	293952.7
14	293775.5

Supplementary Table S7. The QAICs of *df* of natural cubic spline.

df	*QAICS*
3	291121.6
4	291003.0
5	291143.9

5.selecting equal knots of the exposure-response relationship of Temperature on HFMD

The exposure-response relationships were typically modeled using a natural cubic spline. Therefore, we conducted a sensitivity analysis by setting up the exposure-response relationships with natural cubic spline functions, incorporating 2-4 equal knots, and calculating the Quasi-Akaike Information Criterion (QAIC) values (**referenced in Table S8**). After reviewing all the results for the variables, we ultimately selected the spline function with two equal knots.

Supplementary Table S8. The QAICs of equal knots of natural cubic spline.

The number of equal knots	***QAICS***
2	290988.3
3	290994.4
4	291023.0

6.selecting the appropriate weights for the Temperature-Humidity Index (THI)

Considering the humid climate of Yunnan Province, we chose a range for the weight(ω) between 1.1 and 2.5, evaluating using the QAICS. After a thorough review of all variable outcomes, we ultimately settled on ω=1.8. Shown as **Supplementary Table S9.**

Supplementary Table S9. The QAICs of weight(ω) of equal knots of Temperature-Humidity Index (THI).

THI_a	QAICS
$\omega_1 = 1.1$	283877.7
$\omega_2 = 1.2$	283818.5
$\omega_3 = 1.3$	283753.4
$\omega_4 = 1.4$	283687.7
$\omega_5 = 1.5$	283630.5
$\omega_6 = 1.6$	283559.9
$\omega_7 = 1.7$	283544.9
$\omega_8 = 1.8^*$	283540.1
$\omega_9 = 1.9$	283542.3
$\omega_{10} = 2.0$	283549.1
$\omega_{11} = 2.1$	283558.7
$\omega_{12} = 2.2$	283570.0
$\omega_{13} = 2.3$	283582.5
$\omega_{14} = 2.4$	283595.7
$\omega_{15} = 2.5$	283609.3

*The final weight chosen

Supplementary Table S10. Summary statistics of the city-specifc characteristics for the 16 cities in Yunnan, China.

Variables	Number(%)	Mean ± SD	Median (IQR)	Range
HFMD Cases				
Total	684846	188±117	162(98.8,256)	(0,628)
Male	401151(58.58)	110±68.3	96(57,150)	(0,364)
Female	283695(41.42)	78±49.1	67(41,106)	(0,271)
0≤Age< 3 year	437731(63.92)	120±71.3	105(66,163)	(0,383)
3≤Age< 6 years	247115(37.08)	67.7±49.3	56(29,94)	(0,308)
Meteorology Measure				
Mean temperature(℃)	/	16.70±4.74	17.50(12.70,20.90)	(2.54,25.20)
Relative humidity (%)	/	70.55±10.66	72.25(63.30,78.94)	(36.83,92.61)
Wind velocity (m/s)	/	1.85±0.46	1.76(1.49,2.16)	(0.91,3.59)
Sunshine hours (h)	/	5.94±2.59	6.24(3.91,8.13)	(0.00,11.91)
PRE (mm)	/	2.74±4.10	0.91(0.03,3.92)	(0.00,47.15)
PRS (hpa)	/	835.34±3.18	835.26(832.89,837.67)	(826.76,846.31)
Several comprehensive Index				
THI_{a8}	/	21.66±7.21	21.67(15.14,29.06)	(3.15,33.05)
Humidex	/	19.06±7.24	19.65(12.63,26.07)	(0.04,31.00)
Heat index	/	16.30±5.18	17.17(11.88,21.11)	(2.05,25.82)

,***PRE*** precipitation ***PRS*** pressure ***IQR*** interquartile range

Supplementary Table S11. Summary statistics of the city-specifc characteristics for the 16 cities in Yunnan, China.

Area	Wald test	*df*	*P* value
Yunnan	44.700	3	＜0.001
Kunming	43.313	3	＜0.001
Qujing	40.531	3	＜0.001
Yuxi	19.624	3	＜0.001
Baoshan	17.163	3	＜0.001
Zhaotong	14.901	3	0.002
Lijiang	2.857	3	0.224
Pu’er	53.049	3	＜0.001
Lincang	4.838	3	0.184
Chuxiong	88.575	3	＜0.001
Honghe	39.813	3	＜0.001
Wenshan	4.463	3	0.216
Xishuang Banna	21.439	3	＜0.001
Dali	43.694	3	＜0.001
Dehong	32.086	3	＜0.001
Nujiang	7.815	3	0.050
Diqing	5.100	3	0.165

Supplementary Table S12. Exposure-response relationships between THI_{a8} and HFMD for each year from 2010 to 2019 by fitting with time-varying distributed lag nonlinear model (Total)(A).

Period	Low level RR(95%CI)		High level RR(95%CI)	
	P_1	P_{25}	P_{75}	P_{99}
2010	0.842(0.622,1.140)	0.691(0.574,0.833)	1.118(1.047,1.194)	1.068(0.755,1.511)
2011	0.812(0.628,1.050)	0.707(0.602,0.830)	1.118(1.055,1.186)	1.133(0.840,1.529)
2012	0.783(0.634,0.968)	0.722(0.629,0.828)	1.118(1.062,1.178)	1.203(0.934,1.551)
2013	0.755(0.640,0.891)	0.738(0.658,0.828)	1.119(1.069,1.171)	1.280(1.037,1.580)
2014	0.727(0.643,0.822)	0.754(0.686,0.828)	1.119(1.075,1.165)	1.364(1.147,1.622)
2015	0.700(0.641,0.763)	0.769(0.711,0.833)	1.121(1.081,1.163)	1.458(1.258,1.689)
2016	0.674(0.625,0.727)	0.786(0.729,0.847)	1.123(1.084,1.163)	1.560(1.357,1.793)
2017	0.652(0.603,0.707)	0.804(0.743,0.870)	1.123(1.084,1.164)	1.660(1.428,1.930)
2018	0.635(0.580,0.695)	0.826(0.757,0.901)	1.122(1.081,1.164)	1.755(1.469,2.097)
2019	0.617(0.552,0.691)	0.850(0.770,0.937)	1.119(1.074,1.165)	1.846(1.490,2.288)

P_1, 1st percentile of THI_{a8}; P_{25}; 25th percentile of THI_{a8}; P_{75}, 75th percentile of THI_{a8}; P_{99} , 99th percentile of THI_{a8}.

Supplementary Table S13. Exposure-response relationships between THI_{a8} and HFMD for each year from 2010 to 2019 by fitting with time-varying distributed lag nonlinear model (Total)(B).

Period	Low level RR(95%CI)		High level RR(95%CI)	
	$P_{2.5}$	P_5	P_{95}	$P_{97.5}$
2010	0.826(0.616,1.109)	0.801(0.606,1.060)	1.085(0.809,1.456)	1.074(0.774,1.490)
2011	0.801(0.624,1.028)	0.783(0.617,0.993)	1.138(0.883,1.467)	1.135(0.856,1.506)
2012	0.776(0.632,0.953)	0.764(0.628,0.930)	1.194(0.963,1.482)	1.200(0.944,1.524)
2013	0.752(0.639,0.883)	0.746(0.639,0.871)	1.256(1.049,1.502)	1.270(1.041,1.550)
2014	0.727(0.645,0.820)	0.728(0.648,0.818)	1.321(1.139,1.533)	1.348(1.144,1.588)
2015	0.703(0.645,0.766)	0.709(0.651,0.772)	1.394(1.228,1.582)	1.434(1.247,1.648)
2016	0.681(0.631,0.734)	0.692(0.642,0.746)	1.473(1.306,1.660)	1.527(1.338,1.742)
2017	0.662(0.612,0.716)	0.678(0.626,0.733)	1.548(1.361,1.762)	1.617(1.402,1.865)
2018	0.647(0.591,0.708)	0.667(0.611,0.729)	1.619(1.391,1.883)	1.703(1.439,2.014)
2019	0.632(0.566,0.706)	0.657(0.590,0.731)	1.684(1.405,2.019)	1.784(1.457,2.183)

$P_{2.5}$, 2.5th percentile of THI_{a8}; P_5; 5th percentile of THI_{a8}; P_{95}, 95th percentile of THI_{a8}; $P_{97.5}$, 97.5th percentile of THI_{a8}.

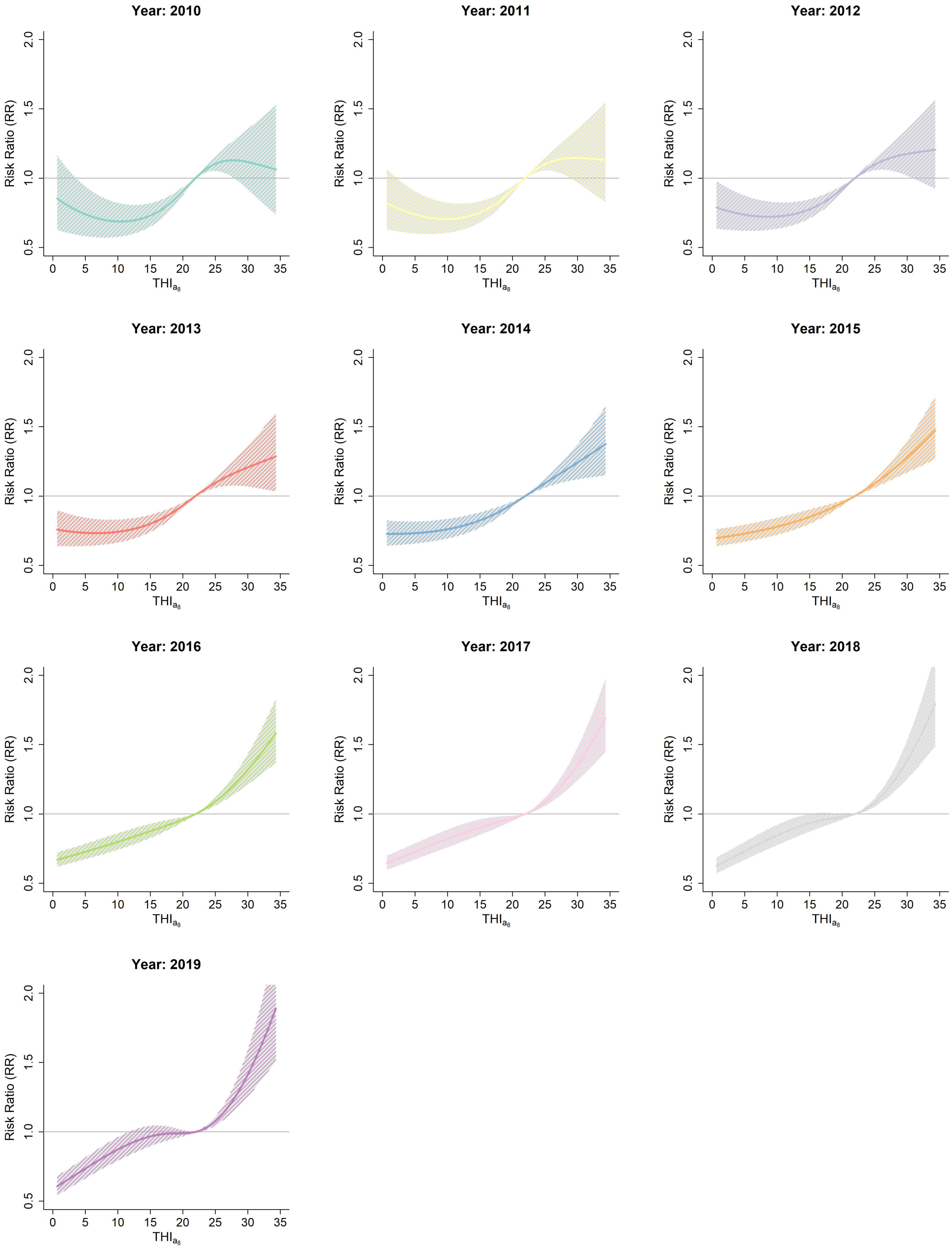

Supplementary Figure S11. Annual Trends in the Association Between THI$_{a8}$ and HFMD in Yunnan Province, China (2010-2019)(Total).

Supplementary Table S14. Exposure-response relationships between THI_{a8} and HFMD for each year from 2010 to 2019 by fitting with time-varying distributed lag nonlinear model (Male)(A).

Period	Low level RR(95%CI)		High level RR(95%CI)	
	P_1	P_{25}	P_{75}	P_{99}
2010	0.867(0.662,1.134)	0.706(0.607,0.820)	1.121(1.048,1.199)	1.098(0.757,1.593)
2011	0.831(0.664,1.040)	0.719(0.602,0.817)	1.121(1.058,1.188)	1.167(0.847,1.607)
2012	0.793(0.662,0.950)	0.730(0.656,0.814)	1.123(1.068,1.181)	1.242(0.950,1.627)
2013	0.754(0.653,0.871)	0.740(0.675,0.811)	1.124(1.075,1.176)	1.323(1.051,1.665)
2014	0.715(0.638,0.803)	0.750(0.691,0.814)	1.126(1.082,1.173)	1.408(1.163,1.706)
2015	0.681(0.618,0.750)	0.761(0.702,0.824)	1.128(1.086,1.171)	1.501(1.281,1.759)
2016	0.653(0.597,0.715)	0.776(0.713,0.845)	1.129(1.090,1.170)	1.605(1.403,1.836)
2017	0.633(0.576,0.695)	0.796(0.725,0.873)	1.130(1.089,1.172)	1.711(1.498,1.953)
2018	0.617(0.557,0.683)	0.818(0.740,0.905)	1.128(1.083,1.175)	1.810(1.537,2.132)
2019	0.603(0.538,0.675)	0.844(0.758,0.939)	1.125(1.074,1.180)	1.914(1.558,2.350)

P_1, 1st percentile of THI_{a8}; P_{25}; 25th percentile of THI_{a8}; P_{75}, 75th percentile of THI_{a8}; P_{99} , 99th percentile of THI_{a8}.

Supplementary Table S15. Exposure-response relationships between THI_{a8} and HFMD for each year from 2010 to 2019 by fitting with time-varying distributed lag nonlinear model (Male)(B).

Period	Low level RR(95%CI)		High level RR(95%CI)	
	$P_{2.5}$	P_5	P_{95}	$P_{97.5}$
2010	0.850(0.655,1.102)	0.824(0.645,1.053)	1.111(0.810,1.522)	1.103(0.777,1.566)
2011	0.818(0.659,1.017)	0.800(0.651,0.982)	1.166(0.889,1.529)	1.166(0.863,1.577)
2012	0.786(0.660,0.936)	0.774(0.656,0.914)	1.227(0.975,1.542)	1.236(0.958,1.595)
2013	0.751(0.652,0.864)	0.746(0.652,0.852)	1.291(1.062,1.569)	1.311(1.055,1.628)
2014	0.716(0.640,0.801)	0.717(0.644,0.799)	1.358(1.153,1.599)	1.389(1.159,1.665)
2015	0.685(0.623,0.753)	0.692(0.631,0.758)	1.429(1.248,1.638)	1.474(1.268,1.713)
2016	0.660(0.604,0.722)	0.660(0.616,0.735)	1.509(1.344,1.694)	1.568(1.380,1.782)
2017	0.643(0.586,0.706)	0.660(0.601,0.724)	1.588(1.417,1.781)	1.664(1.467,1.887)
2018	0.630(0.569,0.697)	0.651(0.589,0.720)	1.662(1.444,1.912)	1.753(1.502,2.046)
2019	0.618(0.553,0.691)	0.644(0.577,0.718)	1.737(1.458,2.069)	1.845(1.520,2.240)

$P_{2.5}$, 2.5th percentile of THI_{a8}; P_5; 5th percentile of THI_{a8}; P_{95}, 95th percentile of THI_{a8}; $P_{97.5}$, 97.5th percentile of THI_{a8}.

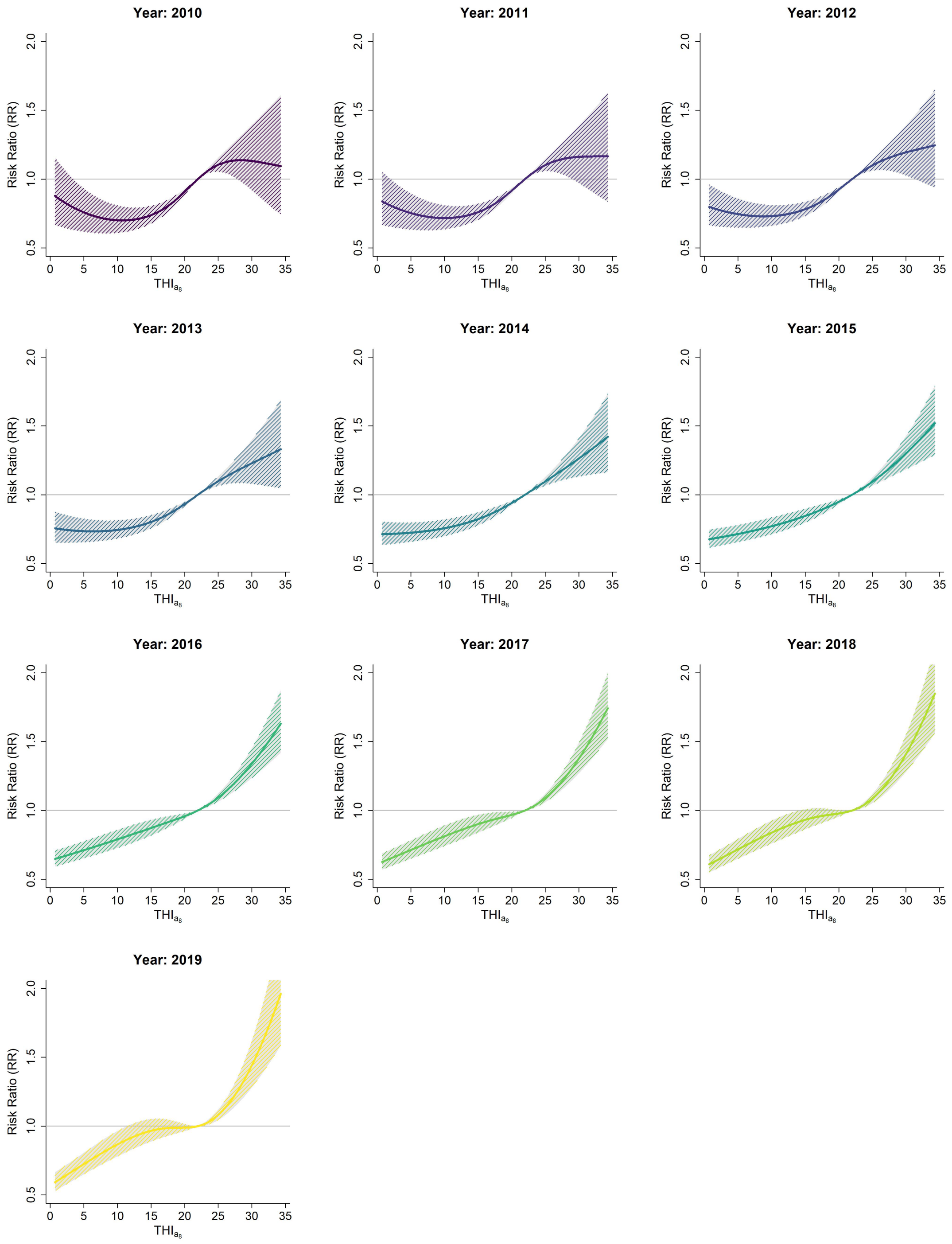

Supplementary Figure S12. Annual Trends in the Association Between THI_{a8} and HFMD in Yunnan Province, China (2010-2019)(Male).

Supplementary Table S16. Exposure-response relationships between THI_{a8} and HFMD for each year from 2010 to 2019 by fitting with time-varying distributed lag nonlinear model (Female)(A).

Period	Low level RR(95%CI)		High level RR(95%CI)	
	P_1	P_{25}	P_{75}	P_{99}
2010	0.831(0.555,1.243)	0.655(0.494,0.867)	1.130(1.045,1.222)	1.038(0.682,1.582)
2011	0.797(0.564,1.125)	0.672(0.526,0.857)	1.132(1.055,1.214)	1.120(0.778,1.611)
2012	0.766(0.574,1.021)	0.690(0.561,0.848)	1.133(1.064,1.206)	1.208(0.886,1.648)
2013	0.738(0.586,0.930)	0.709(0.597,0.842)	1.134(1.072,1.200)	1.303(1.002,1.695)
2014	0.714(0.599,0.851)	0.730(0.636,0.838)	1.135(1.079,1.194)	1.407(1.125,1.760)
2015	0.693(0.612,0.786)	0.753(0.675,0.840)	1.137(1.086,1.191)	1.520(1.246,1.855)
2016	0.670(0.603,0.743)	0.774(0.704,0.851)	1.139(1.090,1.190)	1.647(1.353,2.004)
2017	0.643(0.575,0.720)	0.795(0.722,0.876)	1.139(1.090,1.190)	1.763(1.418,2.191)
2018	0.618(0.537,0.710)	0.818(0.732,0.914)	1.136(1.084,1.190)	1.873(1.449,2.421)
2019	0.593(0.496,0.709)	0.842(0.737,0.961)	1.133(1.076,1.194)	1.996(1.469,2.713)

P_1, 1st percentile of THI_{a8}; P_{25}; 25th percentile of THI_{a8}; P_{75}, 75th percentile of THI_{a8}; P_{99} , 99th percentile of THI_{a8}.

Supplementary Table S17. Exposure-response relationships between THI_{a8} and HFMD for each year from 2010 to 2019 by fitting with time-varying distributed lag nonlinear model (Female)(B).

Period	Low level RR(95%CI)		High level RR(95%CI)	
	$P_{2.5}$	P_5	P_{95}	$P_{97.5}$
2010	0.812(0.547,1.204)	0.783(0.535,1.145)	1.064(0.746,1.516)	1.105(0.705,1.557)
2011	0.783(0.559,1.098)	0.762(0.550,1.056)	1.131(0.832,1.537)	1.124(0.798,1.583)
2012	0.757(0.571,1.003)	0.743(0.566,0.976)	1.202(0.925,1.563)	1.206(0.900,1.616)
2013	0.733(0.584,0.920)	0.726(0.583,0.904)	1.278(1.023,1.597)	1.294(1.001,1.658)
2014	0.713(0.600,0.847)	0.712(0.601,0.842)	1.359(1.123,1.645)	1.389(1.124,1.716)
2015	0.696(0.615,0.787)	0.700(0.620,0.791)	1.447(1.221,1.716)	1.493(1.237,1.801)
2016	0.676(0.610,0.749)	0.686(0.620,0.759)	1.544(1.306,1.826)	1.607(1.335,1.935)
2017	0.653(0.584,0.729)	0.668(0.600,0.744)	1.630(1.356,1.961)	1.712(1.394,2.102)
2018	0.630(0.550,0.722)	0.652(0.571,0.743)	1.711(1.378,2.124)	1.811(1.422,2.305)
2019	0.608(0.511,0.724)	0.635(0.537,0.750)	1.800(1.390,2.330)	1.920(1.439,2.562)

$P_{2.5}$, 2.5th percentile of THI_{a8}; P_5; 5th percentile of THI_{a8}; P_{95}, 95th percentile of THI_{a8}; $P_{97.5}$, 97.5th percentile of THI_{a8}.

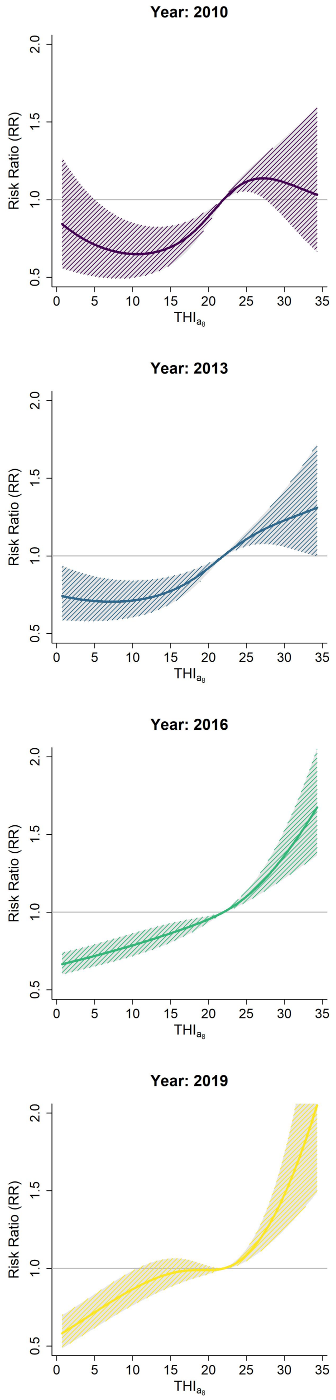

Supplementary Figure S13. Annual Trends in the Association Between THI_{a8} and HFMD in Yunnan Province, China (2010-2019)(Female).

Supplementary Table S18. Exposure-response relationships between THI_{a8} and HFMD for each year from 2010 to 2019 by fitting with time-varying distributed lag nonlinear model (0≤Age＜3)(A).

Period	Low level RR(95%CI)		High level RR(95%CI)	
	P_1	P_{25}	P_{75}	P_{99}
2010	0.965(0.690,1.349)	0.720(0.589,0.879)	1.182(1.094,1.276)	1.394(0.964,2.014)
2011	0.905(0.679,1.208)	0.725(0.610,0.862)	1.173(1.097,1.254)	1.413(1.029,1.941)
2012	0.849(0.667,1.080)	0.731(0.631,0.846)	1.164(1.098,1.233)	1.434(1.098,1.873)
2013	0.796(0.655,0.967)	0.736(0.652,0.831)	1.156(1.100,1.214)	1.458(1.172,1.814)
2014	0.745(0.642,0.866)	0.741(0.671,0.818)	1.149(1.101,1.198)	1.489(1.252,1.770)
2015	0.697(0.623,0.780)	0.745(0.686,0.810)	1.143(1.102,1.186)	1.533(1.340,1.753)
2016	0.655(0.598,0.717)	0.753(0.697,0.812)	1.137(1.099,1.177)	1.578(1.401,1.777)
2017	0.622(0.569,0.679)	0.765(0.707,0.827)	1.129(1.090,1.170)	1.614(1.424,1.828)
2018	0.594(0.539,0.656)	0.780(0.716,0.850)	1.119(1.076,1.164)	1.642(1.417,1.903)
2019	0.568(0.506,0.638)	0.798(0.725,0.878)	1.108(1.059,1.160)	1.668(1.393,1.997)

P_1, 1st percentile of THI_{a8}; P_{25}; 25th percentile of THI_{a8}; P_{75}, 75th percentile of THI_{a8}; P_{99} , 99th percentile of THI_{a8}.

Supplementary Table S19. Exposure-response relationships between THI_{a8} and HFMD for each year from 2010 to 2019 by fitting with time-varying distributed lag nonlinear model (0≤Age＜3)(B).

Period	Low level RR(95%CI)		High level RR(95%CI)	
	$P_{2.5}$	P_5	P_{95}	$P_{97.5}$
2010	0.940(0.679,1.301)	0.902(0.662,1.227)	1.363(0.996,1.865)	1.382(0.976,1.957)
2011	0.887(0.671,1.172)	0.859(0.659,1.118)	1.376(1.050,1.803)	1.399(1.037,1.888)
2012	0.837(0.663,1.056)	0.817(0.655,1.019)	1.389(1.106,1.745)	1.417(1.101,1.824)
2013	0.789(0.654,0.952)	0.777(0.651,0.929)	1.405(1.166,1.694)	1.438(1.170,1.768)
2014	0.743(0.642,0.859)	0.739(0.644,0.848)	1.427(1.230,1.655)	1.465(1.124,1.726)
2015	0.699(0.627,0.779)	0.702(0.633,0.778)	1.459(1.299,1.638)	1.504(1.324,1.709)
2016	0.660(0.604,0.721)	0.669(0.615,0.729)	1.491(1.345,1.653)	1.544(1.379,1.729)
2017	0.630(0.578,0.687)	0.645(0.593,0.701)	1.515(1.360,1.688)	1.576(1.400,1.774)
2018	0.606(0.550,0.667)	0.625(0.569,0.686)	1.534(1.350,1.742)	1.601(1.392,1.841)
2019	0.583(0.520,0.652)	0.606(0.544,0.676)	1.549(1.327,1.809)	1.622(1.368,1.924)

$P_{2.5}$, 2.5th percentile of THI_{a8}; P_5; 5th percentile of THI_{a8}; P_{95}, 95th percentile of THI_{a8}; $P_{97.5}$, 97.5th percentile of THI_{a8}.

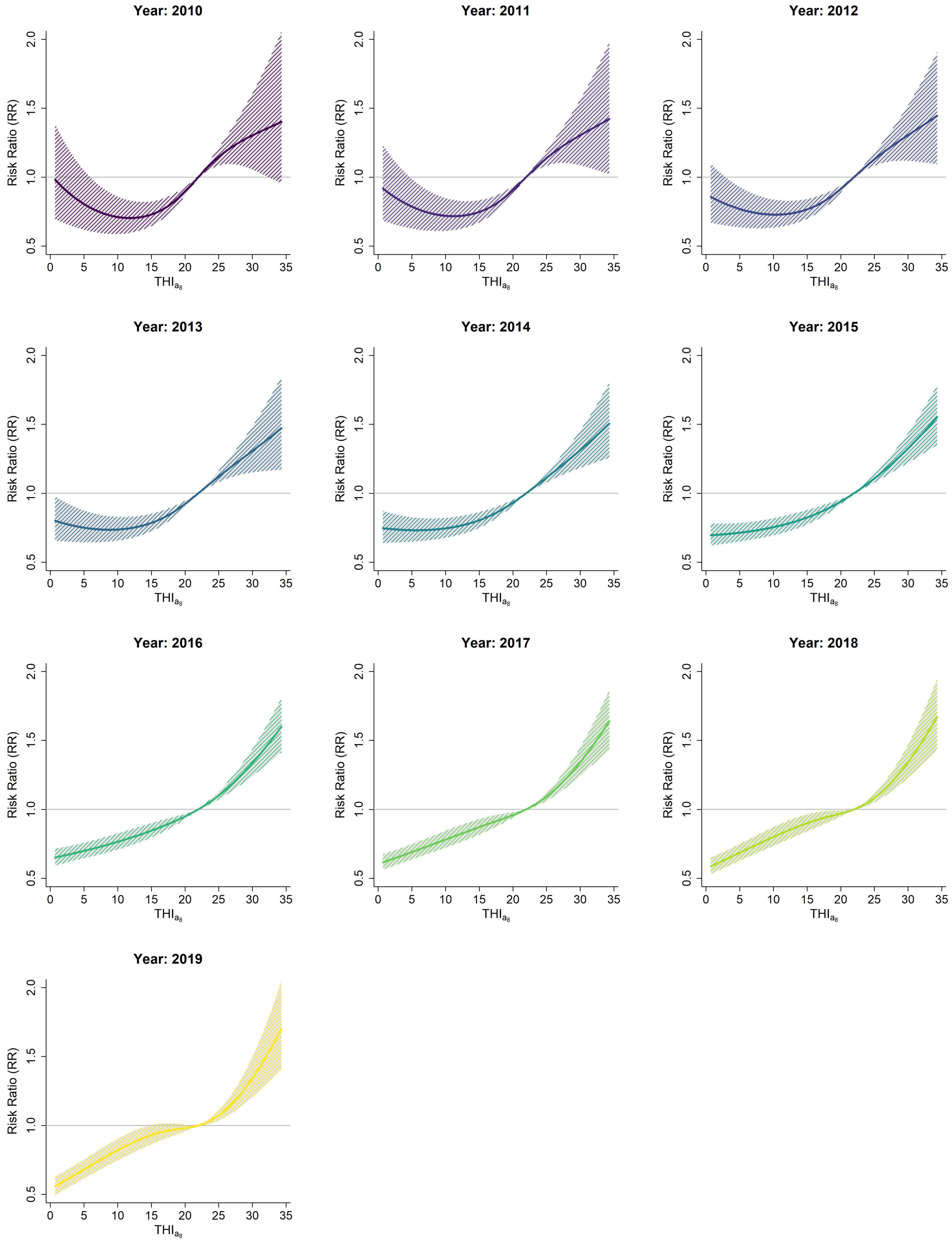

Supplementary Figure S14. Annual Trends in the Association Between THI$_{a8}$ and HFMD in Yunnan Province, China (2010-2019) (0⩽Age＜3).

Supplementary Table S20. Exposure-response relationships between THI_{a8} and HFMD for each year from 2010 to 2019 by fitting with time-varying distributed lag nonlinear model (3≤Age<6)(A).

Period	Low level RR(95%CI)		High level RR(95%CI)	
	P_1	P_{25}	P_{75}	P_{99}
2010	0.709(0.510,0.986)	0.631(0.501,0.793)	1.041(0.977,1.109)	0.704(0.463,1.069)
2011	0.710(0.537,0.937)	0.662(0.545,0.805)	1.052(1.001,1.112)	0.809(0.565,1.158)
2012	0.709(0.564,0.891)	0.696(0.592,0.819)	1.064(1.014,1.115)	0.931(0.685,1.264)
2013	0.707(0.588,0.849)	0.732(0.642,0.835)	1.075(1.031,1.121)	1.071(0.824,1.391)
2014	0.703(0.611,0.810)	0.768(0.693,0.851)	1.087(1.044,1.131)	1.232(1.001,1.552)
2015	0.698(0.625,0.779)	0.803(0.741,0.871)	1.099(1.055,1.147)	1.424(1.140,1.778)
2016	0.690(0.623,0.763)	0.839(0.779,0.903)	1.113(1.064,1.165)	1.642(1.301,2.073)
2017	0.681(0.611,0.759)	0.872(0.804,0.945)	1.126(1.073,1.183)	1.879(1.449,2.436)
2018	0.679(0.595,0.776)	0.912(0.829,0.996)	1.138(1.078,1.201)	2.136(1.577,2.892)
2019	0.680(0.570,0.813)	0.958(0.851,1.079)	1.148(1.080,1.219)	2.426(1.689,3.484)

P_1, 1st percentile of THI_{a8}; P_{25}; 25th percentile of THI_{a8}; P_{75}, 75th percentile of THI_{a8}; P_{99} , 99th percentile of THI_{a8}.

Supplementary Table S21. Exposure-response relationships between THI_{a8} and HFMD for each year from 2010 to 2019 by fitting with time-varying distributed lag nonlinear model (3≤Age<6)(B).

Period	Low level RR(95%CI)		High level RR(95%CI)	
	$P_{2.5}$	P_5	P_{95}	$P_{97.5}$
2010	0.699(0.506,0.965)	0.684(0.501,0.934)	0.762(0.537,1.082)	0.725(0.490,1.074)
2011	0.703(0.535,0.923)	0.692(0.532,0.901)	0.854(0.632,1.154)	0.825(0.589,1.156)
2012	0.705(0.563,0.883)	0.700(0.563,0.870)	0.959(0.742,1.240)	0.941(0.706,1.255)
2013	0.706(0.590,0.845)	0.706(0.593,0.840)	1.076(0.864,1.341)	1.073(0.839,1.372)
2014	0.706(0.615,0.811)	0.711(0.622,0.814)	1.208(0.994,1.468)	1.223(0.984,1.520)
2015	0.704(0.632,0.784)	0.715(0.644,0.793)	1.361(1.127,1.644)	1.400(1.135,1.726)
2016	0.699(0.633,0.771)	0.715(0.650,0.786)	1.531(1.256,1.867)	1.600(1.284,1.993)
2017	0.693(0.624,0.770)	0.714(0.645,0.790)	1.711(1.373,2.133)	1.814(1.420,2.317)
2018	0.695(0.611,0.790)	0.720(0.638,0.814)	1.902(1.472,2.458)	2.045(1.537,2.721)
2019	0.699(0.589,0.830)	0.730(0.621,0.858)	2.111(1.556,2.865)	2.303(1.638,3.238)

$P_{2.5}$, 2.5th percentile of THI_{a8}; P_5; 5th percentile of THI_{a8}; P_{95}, 95th percentile of THI_{a8}; $P_{97.5}$, 97.5th percentile of THI_{a8}.

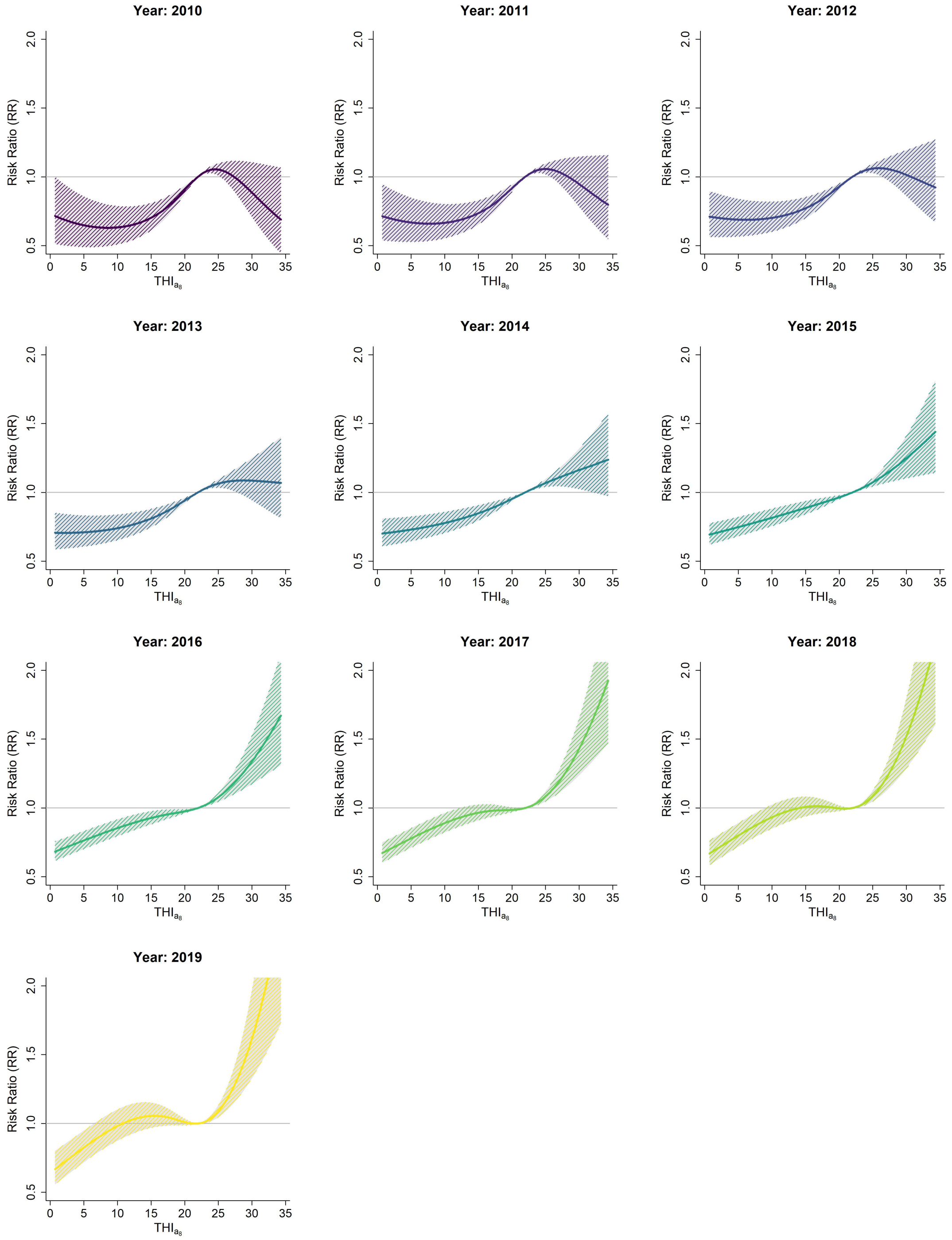

Supplementary Figure S15. Annual Trends in the Association Between THI_{a8} and HFMD in Yunnan Province, China (2010-2019) (3≤Age<6).
